# Controlling amphiphilic copolymer self-assembly morphologies based on macrocycle/anion recognition and nucleotide-induced payload release†
†Electronic supplementary information (ESI) available. See DOI: 10.1039/c6sc01851c


**DOI:** 10.1039/c6sc01851c

**Published:** 2016-05-24

**Authors:** Xiaofan Ji, Hu Wang, Yang Li, Danyu Xia, Hao Li, Guping Tang, Jonathan L. Sessler, Feihe Huang

**Affiliations:** a State Key Laboratory of Chemical Engineering , Center for Chemistry of High-Performance & Novel Materials , Department of Chemistry , Zhejiang University , Hangzhou 310027 , P. R. China . Email: fhuang@zju.edu.cn ; Fax: +86-571-8795-3189 ; Tel: +86-571-8795-3189; b Department of Chemistry , The University of Texas at Austin , 105 East 24th Street, Stop A5300 , Austin , Texas 78712 , USA . Email: sessler@cm.utexas.edu; c Institute for Supramolecular and Catalytic Chemistry , Shanghai University , Shanghai 200444 , China

## Abstract

We report here a new approach for creating diversiform copolymer-derived self-assembly morphologies that relies on macrocycle/anion recognition in aqueous media. This approach exploits the anion binding features of a water-soluble form of the so-called ‘Texas-sized’ molecular box. When this tetracationic receptor is added to an aqueous solution of an amphiphilic copolymer bearing tethered carboxylate anion substituents, binding occurs to form a macrocycle/polymer complex. As the concentration of the box-like receptor increases, the relative hydrophilic fraction of the copolymer complex likewise increases. This leads to changes in the overall morphology of the self-assembled ensemble. The net result is an environmentally controllable system that mimics on a proof-of-concept level the structural evolution of organelles seen in living cells. The macrocycle/anion interactions respond in differing degrees to three key biological species, namely ATP, ADP, and AMP, which may be used as “inputs” to induce disassembly of these vehicles. As a result of this triggering and the nature of the morphological changes induced, the present copolymer system is capable of capturing and releasing in a controlled manner various test payloads, including hydrophobic and hydrophilic fluorophores. The copolymer displays a low inherent cytotoxicity as inferred from cell proliferation assays involving the HUVEC and HepG2 cell lines.

## Abstract 2

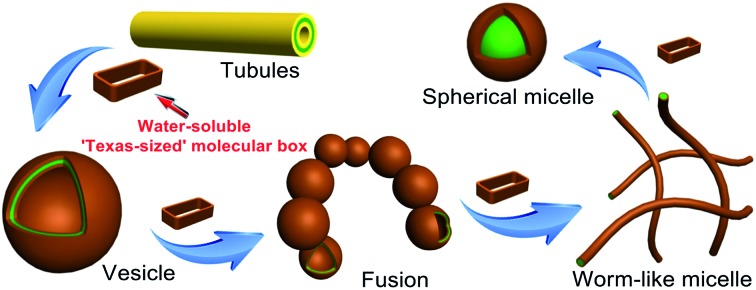
We create polymeric self-assembly morphologies by exploiting the anion binding features of the so-called ‘Texas-sized’ molecular box.

## Introduction

The membrane-encapsulated organelles are not uniform in size and shape. Rather they are characterized by a tremendous diversity of structures.^1^ For example, spheres, tubular networks, systems of interconnected sheets and tubules, and stacks of perforated sheets are observed for lysosomes (peroxisomes), mitochondria, the endoplasmic reticulum and the Golgi apparatus, respectively.^2^ Moreover, changes in these shapes often occur during cell movement, division, and growth, and are often the result of the membrane-associated fusion of vesicles.^2^ The complexity of these natural processes provides an incentive to prepare and study synthetic systems that might allow the fundamental relationship between environment and structure to be better understood. Materials that have proved attractive in this latter regard are amphiphilic copolymers.

Amphiphilic copolymers, polymeric systems containing hydrophilic/hydrophobic domains, have attracted attention across a diversity of fields, including polymer science, supramolecular chemistry, and biomedicine, in part because they can self-assemble into diversiform structures in water, including vesicles, lamellae, bicontinuous structures, cylindrical micelles, disk-like micelles, spherical micelles and other complex or hierarchical aggregates.^3^ Owing to the good mechanical and physical properties resulting from the use of covalent polymeric backbones, polymer aggregates derived from amphiphilic copolymers generally display higher stability and durability than most small molecule aggregates.^3^ This has made amphiphilic copolymers attractive candidates for exploring the determinants that underlie the shape transformations observed in the case of naturally occurring vesicles and organelles. Traditionally, the morphologies of amphiphilic polymer aggregates can be controlled by tuning the ratio of the hydrophilic/hydrophobic portions within the copolymer. However, such modifications require sophisticated polymer design, dedicated polymer syntheses, and precise control over processing.^4^ Recently, dynamic and reversible host–guest interactions have been proposed as a very important means of adjusting the hydrophilic/hydrophobic balance within amphiphilic copolymers and hence controlling the morphologies produced *via* polymer self-assembly.^5^,^6^ To date, these efforts have for the most part relied on macrocycle-based host–guest interactions involving cationic or neutral guests. Macrocycle/anion recognition has rarely been exploited as a means of adjusting polymer structure and behaviour.^5^,^6^ However, anions are ubiquitous within the broader environment and are intimately involved in many biological processes.^7^ Therefore, we consider it important to develop new approaches wherein anionic guest-based host–guest interactions in water are used to control amphiphilic polymer self-assembly and the associated morphologies. Of particular interest would be systems that exploit biologically important anions as controlling elements. Here, we report such a system, wherein a competition between carboxylate anion and phosphorylated nucleotide recognition is used to promote self-assembly, payload capture, and controlled release.

In 2010, Sessler and coworkers reported a first-generation ‘Texas-sized’ molecular box, cyclo[2](2,6-di(1H-imidazole-1-yl)pyridine)[2](1,4-dimethylenebenzene), that could bind the mono-terephthalate anion to form a host–guest complex.^8^ This tetracationic macrocycle was designed to provide a larger alternative to the original blue box, (cylcobis(paraquat-*p*-phenylene) or CBPQT^4+^), developed by Stoddart and co-workers.^9^ Compared to the blue box, the Texas-sized box is conformationally flexible. It can adjust its shape so as to accommodate guest molecules with different sizes, charges, and shapes; it even displays different conformations with the same guest molecule.^8^ To date, studies of the Texas-sized box and its anion host–guest interactions have been carried out in organic solvents. This is an inherent limitation in the context of controlling amphiphilic polymer morphologies. To address this latter shortcoming, we have now created a water-soluble form of the Texas-sized molecular box and detail here both its anion host–guest interactions in water and its use in controlling the self-assembly and morphologies of amphiphilic polymer aggregates.

**Scheme 1:**
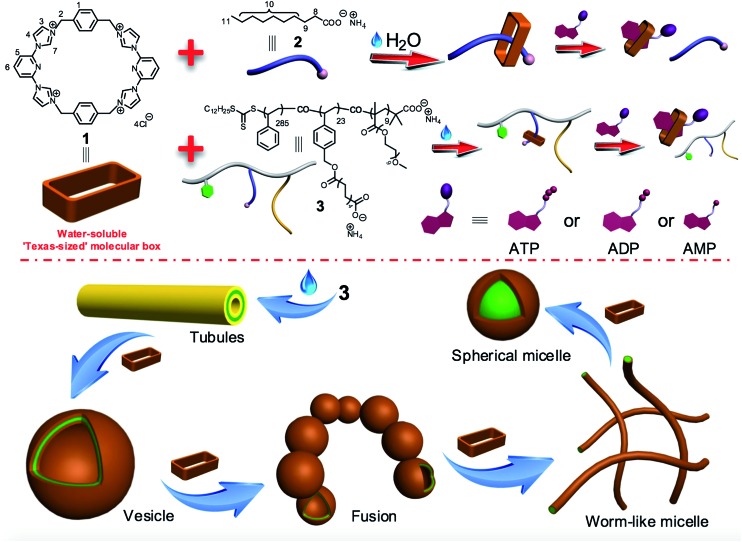
Chemical structures of **1**, **2**, and **3**, cartoon representation of biological signals-responsive macrocycle/anion interactions, and schematic views of the diverse assemblies formed from **3** and **1** as the result of targeted molecular recognition.

As shown in Scheme 1, the water-soluble Texas-sized molecular box **1** (preparation detailed below) can bind ammonium decanoate **2** to form a stable 1 : 1 complex in water. It was thus expected to interact well with a polymer containing pendant carboxylic acid moieties. Copolymer **3** contains domains bearing carboxylate anions and was thus chosen as oligomeric target for **1**. This copolymer consists of three types of repeating units derived from three kinds of monomers: hydrophobic styrene, a hydrophilic oligo(ethylene glycol) methacrylate (OEGMA), and a styrene derivative bearing the salt form of a long chain carboxylic acid analogous to **2**. In water, copolymer **3** can self-assemble into tube structures. On the other hand, when macrocycle **1** is added into aqueous solutions of **3**, binding to the carboxylate-containing repeating units was expected, thus changing in a monotonic manner the hydrophilic–hydrophobic balance of the overall macrocycle/copolymer complex. Since the self-assembly morphology of polymer aggregates is affected by the polymer amphiphilicity,^10^ this macrocycle/anion host–guest recognition was expected to control the morphology of the resulting polymer aggregates.

The micelles and vesicles derived from macrocycle **1** and copolymer **3** are further attractive because in due course they may see application as delivery vehicles capable of, *e.g.*, transporting various small molecules (“payloads”) to cells and allowing for their controlled release.^8^,^11^ For these latter putative intracellular applications, the use of intracellular biological signals (“inputs”) is attractive since it might allow control over drug biodistributions in diseased cells and avoid cellular damage induced by the over-accumulation of an inherently toxic chemical agent.^12^ As detailed below, the macrocycle/anion interactions involving **1** respond to specific intracellular biological signals, including adenosine-5′-triphosphate (ATP),^13^ adenosine-5′-diphosphate (ADP), and adenosine-5′-monophosphate (AMP). This interaction, whose effectiveness depends on the degree of nucleotide phosphorylation, can be used to induce vesicle disassembly. The net result is that an anion-responsive copolymer-receptor system may be used to capture and release cytotoxic and fluorescent payloads using biologically relevant triggers, specifically adenosine nucleotides. ATP levels are often considerably elevated, by orders of magnitude, in cancer cells as compared to normal cells.13*c* Constructs that are able to transport chosen payloads and then release them selectively in the presence of ATP might thus have a role to play in the tumour-targeted delivery of diagnostic and therapeutic agents.^13^

## Experimental

### Synthesis of the water-soluble Texas-sized molecular box **1**

**Scheme 2:**
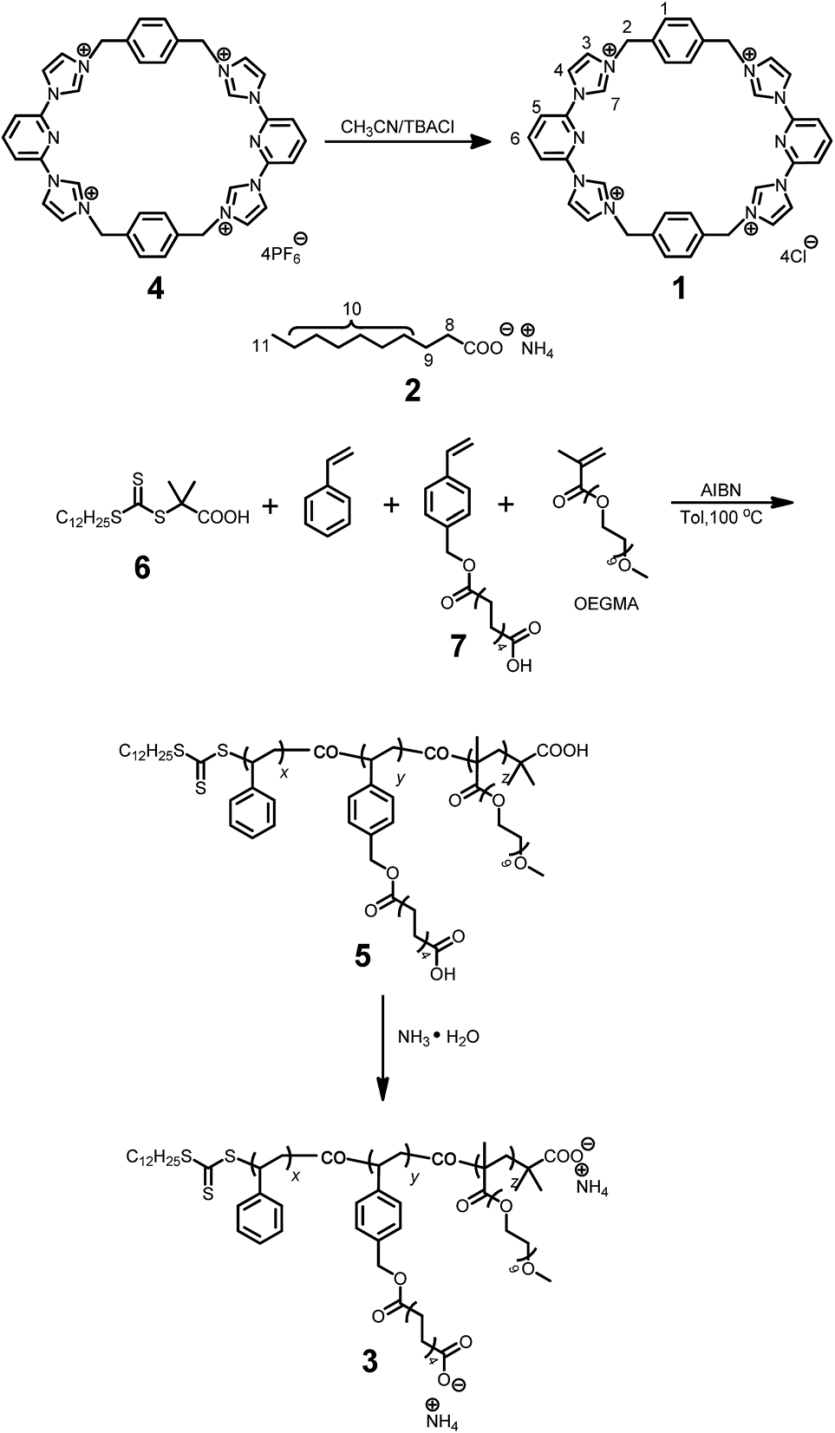
Syntheses of **1** and **3**.

As shown in Scheme 2, the water-soluble macrocycle **1** (tetracation·4Cl^–^) was prepared from its tetrahexafluorophosphate salt precursor (tetracation·4PF_6_^–^) through an ion exchange reaction. Excess tetrabutylammonium chloride (TBACl) was added to a solution of **4** (0.242 g, 0.200 mM) in CH_3_CN. This led to formation of a precipitate, which was collected by filtration and washed by CH_3_CN to afford **1** (0.139 g, 0.180 mM, 90%) as a white solid. Mp > 250 °C. The ^1^H NMR spectrum of **1** is shown in Fig. S1.†

^1^H NMR (400 MHz, D_2_O, 298 K) *δ* (ppm): 8.29 (2H, *J* = 8.12 Hz, t), 8.14 (4H, *J* = 1.88 Hz, d), 7.87 (4H, *J* = 8.12 Hz, d), 7.68 (4H, *J* = 1.80 Hz, d), 7.37 (8H, s), 5.47 (8H, s). The ^13^C NMR spectrum of **1** is shown in Fig. S2.† ^13^C NMR (125 MHz, DMSO, 298 K) *δ* (ppm): 148.13, 147.50, 137.02, 131.69, 126.66, 122.51, 117.62, 55.74. The LRESIMS is shown in Fig. S3:† *m*/*z* 221.76 [M – 3Cl]^3+^ (100%), 350.12 [M – 2Cl]^2+^ (50%). HRESIMS: *m*/*z* calcd for [M – 2Cl]^2+^ C_38_H_34_Cl_2_N_10_, 350.1167; found 350.1170; error –0.9 ppm. Other data for **1** are given in the ESI (Fig. S4–S7†).

### Synthesis of polymer **5**

A typical RAFT polymerization was performed using standard Schlenk techniques as follows: 2-dodecylsulfanylthiocarbonylsulfanyl-2-methylpropionic acid **6** (5.00 mg, 0.0143 mmol) as the chain transfer agent (CTA), ammonium decanoate-containing monomer **7** (220 mg, 0.691 mmol), and AIBN (2.30 mg, 0.0140 mmol) were added to a 20 mL Schlenk flask equipped with a stirring bar. This flask was then capped with a rubber septum and deoxygenated by degassing and backfilling with nitrogen three separate times. Then, this flask was charged with toluene (1.00 mL), styrene (0.790 mL, 7.18 mmol), and oligo(ethylene glycol) methacrylate (OEGMA, 0.140 mL, 0.434 mmol) using separate syringes. The mixture was degassed *via* three freeze–evacuate–thaw cycles. The reaction was allowed to proceed under nitrogen at 100 °C for 12 h. The reaction mixture was cooled as needed by liquid nitrogen. After being diluted by THF, the reaction mixture was poured into a beaker containing cold methanol (300 mL) with magnetic stirring. The precipitated solid was collected by vacuum filtration, and washed with methanol several times. This dissolution–precipitation procedure was repeated three times and the yellow solid collected at the end of the final cycle was dried *in vacuo* to give polymer **5** (0.625 g, 58%). The ^1^H NMR spectrum of polymer **5** is shown in Fig. S12.† ^1^H NMR (400 MHz, CDCl_3_, 298 K) *δ* (ppm): 7.24–6.87 (302H, Ar, m), 6.86–6.05 (200H, Ar, m), 5.24–4.85 (15H, Ar-CH_2_O, s), 3.87–2.72 (115H, OCH_2_CH_2_O, m), 2.50–0.74 (550H, main chain and alkyl-chain moieties, m). *M*_n,GPC_ = 41.5 kDa, *M*_w,GPC_ = 47.0 kDa, PDI = 1.13. The ratio of *x*/*y*/*z* is 54.4/4.4/1.7, as calculated from integrations of the protons present in the Ar, Ar-CH_2_O, and OCH_2_CH_2_O subunits, respectively. From the *M*_n_ value and this *x*/*y*/*z* ratio, the values of *x*, *y*, and *z* were calculated to be 285, 23 and 9, respectively.

### Synthesis of polymer **3**

A mixture of **5** (100 mg, 0.00241 mmol) and excess NH_3_·H_2_O (50.0 mL, 14.0 M) was stirred at room temperature for 6 h. The solvent was evaporated under reduced pressure and the residue was dried in vacuum. The product was obtained as a white solid (102 mg, 100%). The ^1^H NMR spectrum of polymer **3** is shown in Fig. S13.†

^1^H NMR (400 MHz, CDCl_3_, 298 K) *δ* (ppm): 7.24–6.85 (302H, Ar, m), 6.83–6.28 (200H, Ar, m), 5.34–4.78 (15H, Ar-CH_2_O, s), 3.73–3.26 (120H, OCH_2_CH_2_O, m), 2.56–0.66 (503H, main chain and alkyl-chain moieties, m).

### Evaluation of the cytotoxicity of the samples in HepG2/HUVEC cells

A standard 3-(4′,5′-dimethylthiazol-2′-yl)-2,5-diphenyl tetrazolium bromide (MTT) assay was used to evaluate the cytotoxicity of the samples in HepG2/HUVEC cells. HepG2/HUVEC cells were seeded in 96-well plates and cultured at 37 °C in a 5% CO_2_ humidified atmosphere for 18 h. Samples with varying concentrations were added to each well, and the cells were added to MTT after being incubated for another 24 h. The absorbance of the solution was measured using a Bio-Rad model 550 microplate reader at 570 nm. Cell viability (%) was equal to (*A*_sample_/*A*_control_) × 100, where *A*_sample_ and *A*_control_ denote the absorbances of the sample well and control well, respectively. Experiments were repeated four times.

### Confocal laser scanning microscopy

Polymer aggregates loaded with FITC or DOX were observed by confocal microscopy using HepG2 cells. The cells were seeded on 24-well plates at 2 × 10^4^ cells per well in 800 μL of RPMI-1640 medium containing 10% FBS and were allowed to grow for 18 h. The medium was replaced by 600 μL of serum-free culture media containing solutions of the samples (80 μg mL^–1^). After 4 h, the cells were rinsed and fixed with fresh 4% paraformaldehyde, and then the cells were treated with DAPI for 5 min. The confocal images were acquired on a confocal scanning laser microscope (CLSM, Radiance 2100, Bio-Rad). The excitation wavelengths were 488 nm for FITC and 543 nm for DOX, respectively.

### Loading and release experiments

DOX (2.00 mg, 3.45 μmol) was dissolved in DMSO (1.0 mL) in the presence of triethylamine (10 μL, 71.7 mmol). After it was completely dissolved, the solution was mixed with an aqueous solution of the micelle (10 mL, 42.0 mg of **3** and 17.7 mg of **1**), and the resulting solution was stirred at room temperature for 2 h. The solution was then transferred into a dialysis tube (MWCO: 2000 Da) and dialyzed against DMSO at room temperature for 12 h. During this time, the dialysis medium was exchanged three times. The solution was then freeze-dried to yield a red powder (59.9 mg) consisting of micelle/DOX. Micelle/DOX (2.00 mg), dissolved in 50 mL of phosphate-buffered saline (PBS) (pH = 7.4), was incubated at 37 °C in dialysis bags with mild stirring. At predefined time points, 1.00 mL of the sample was taken out, and the amount of released DOX induced by exposure to different biological inputs was determined using fluorescence spectroscopy.

FITC (2.00 mg, 5.14 μmol) was dissolved in ethanol (2.0 mL). After it was completely dissolved, the solution was mixed with the vesicle aqueous solution (10 mL, 42.0 mg of **3** and 3.85 mg of **1**), and the resulted solution was stirred at room temperature for 2 h. The solution was then transferred into a dialysis tube (MWCO: 2000 Da) and dialyzed against DMSO at room temperature for 12 h. During this time, the dialysis medium was changed out three times. The solution was then freeze-dried to yield a red powder (46.1 mg) consisting of vesicle/FITC. This vesicle/FITC powder (2.00 mg), dissolved in 50 mL of phosphate-buffered saline (PBS) (pH = 7.4), was incubated at 37 °C in dialysis bags with mild stirring. At predefined time points, 1.00 mL of the sample was taken out, and the amount of released FITC due to different biological triggers was determined by fluorescence spectroscopy.

## Results and discussion

### Host–guest interactions between **1** and **2** in water

**Fig. 1:**
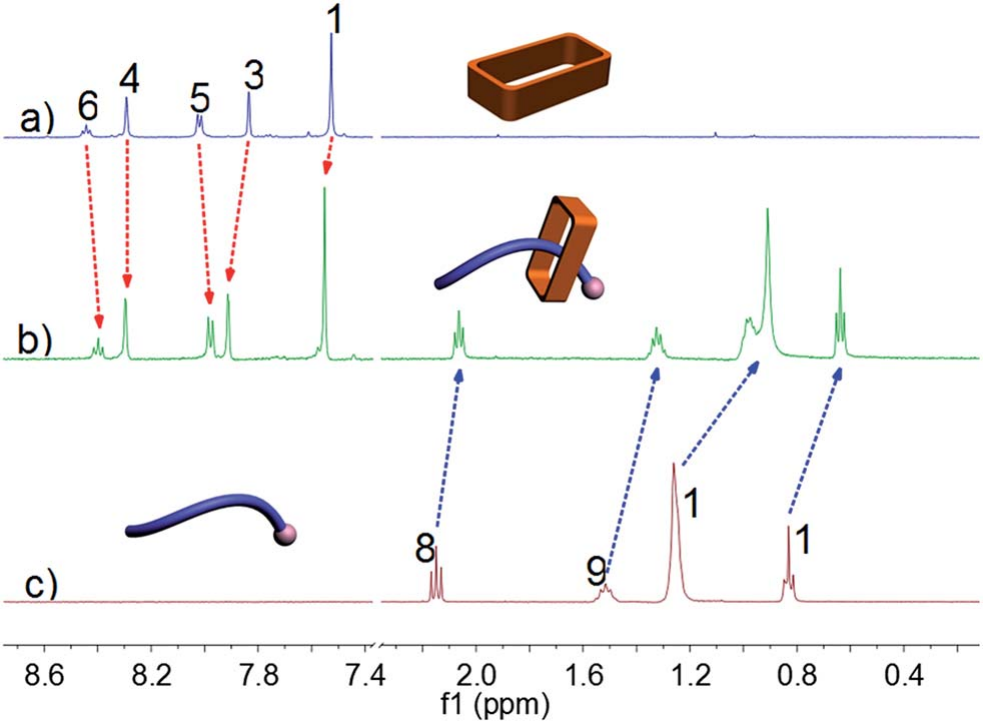
Partial ^1^H NMR spectra (500 MHz, D_2_O, 298 K): (a) 3.00 mM **1**; (b) **1** and **2** (3.00 mM for each); (c) 3.00 mM **2**.

**Fig. 2:**
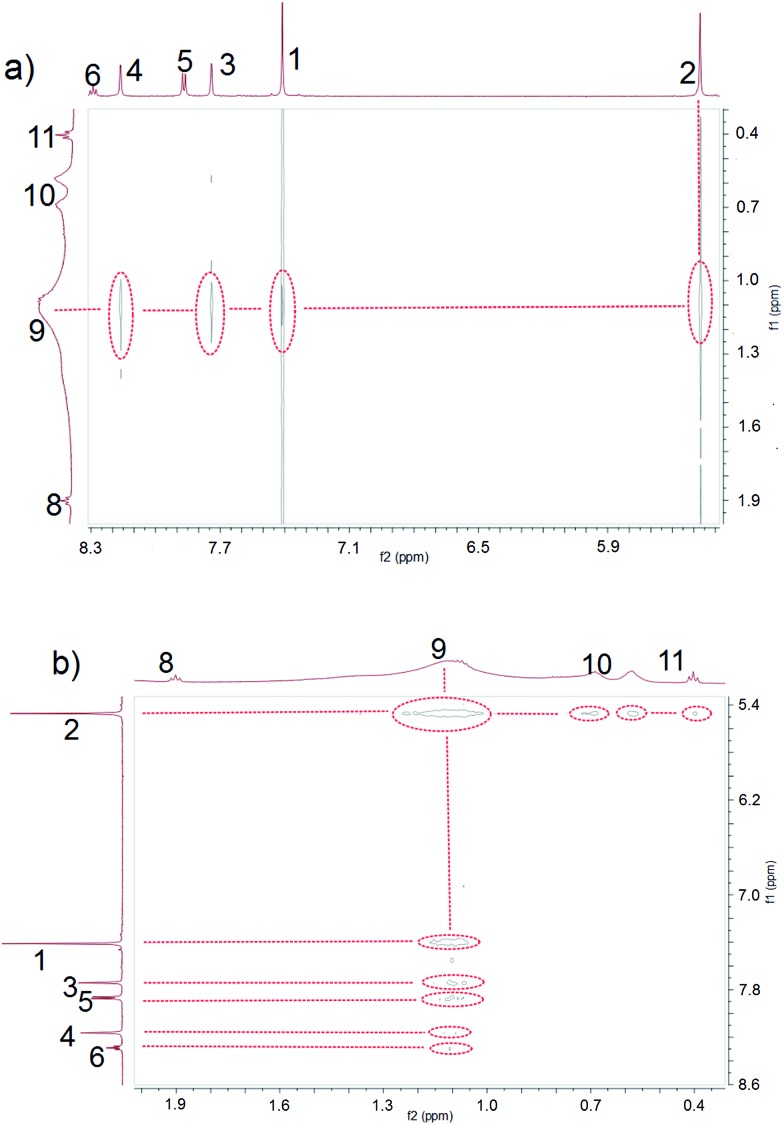
Expanded views of the NOESY NMR spectra (600 MHz, D_2_O, 298 K) of **1** and **2** (each at 3.00 mM).

Ammonium decanoate **2** was chosen as a model substrate to investigate the macrocycle/anion host–guest interactions in water. The interactions between **1** and **2** were first studied by proton NMR spectroscopy. As can be seen from an inspection of Fig. 1, the chemical shifts of diagnostic protons on **1** and **2** undergo substantial changes after the two species are mixed in D_2_O. The resonances associated with protons H_1_ and H_3_ of **1** shift downfield upon mixing, while those for protons H_5_ and H_6_ shift to higher field (spectra a and b in Fig. 1). All the protons (H_8_–H_11_) of **2** undergo upfield shifts (spectra b and c in Fig. 1). In addition, a two-dimensional NOESY NMR spectroscopic analysis carried out using an equimolar solution of **1** and **2** in D_2_O revealed correlation signals between proton H_9_ of guest **2** and protons H_1_–H_6_ of macrocycle **1**, as well as between proton H_2_ of macrocycle **1** and protons H_9_–H_11_ of guest **2** (Fig. 2 and ESI, Fig. S8†).

More quantitative insights came from a ^1^H NMR spectroscopic titration wherein increasing aliquots of **2** were added into a 0.500 mM solution of **1** in water (ESI, Fig. S9†). Addition of 1.0 equiv. of **2** resulted in an upfield shift of 0.10 ppm for proton H_3_ of **1**. However, little appreciable change in this signal was seen when more than 1.0 equiv. of **2** was added. This finding was considered consistent with the formation of a 1 : 1 complex between macrocycle **1** and ammonium decanoate **2** in water. Further support for the proposed 1 : 1 stoichiometry came from a molar ratio plot (ESI, Fig. S11†). A nonlinear curve-fitting analysis (ESI, Fig. S10†) allowed an association constant (*K*_a_) of (2.50 ± 0.38) × 10^5^ M^–1^ to be calculated for **1** ⊃ **2** in water.

### Controlling amphiphilic copolymer self-assembly morphologies using macrocycle **1**

**Fig. 3:**
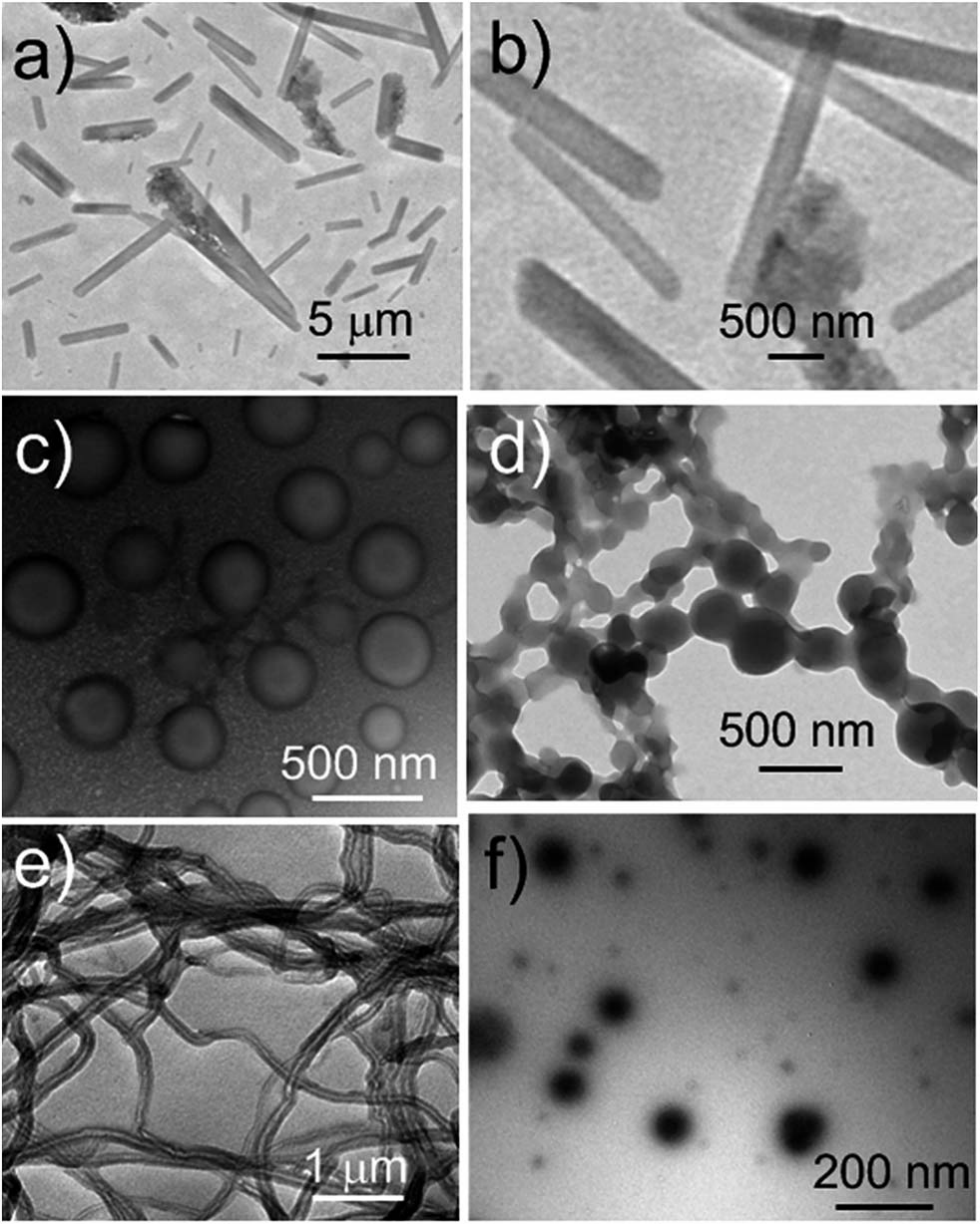
TEM images of the aggregates formed from copolymer **3** (0.100 mM) upon the addition of different amounts of **1** (mM): (a) 0; (b) enlarged view of the image appearing in (a); (c) 0.500; (d) 1.00; (e) 1.50; (f) 2.30.

**Fig. 4:**
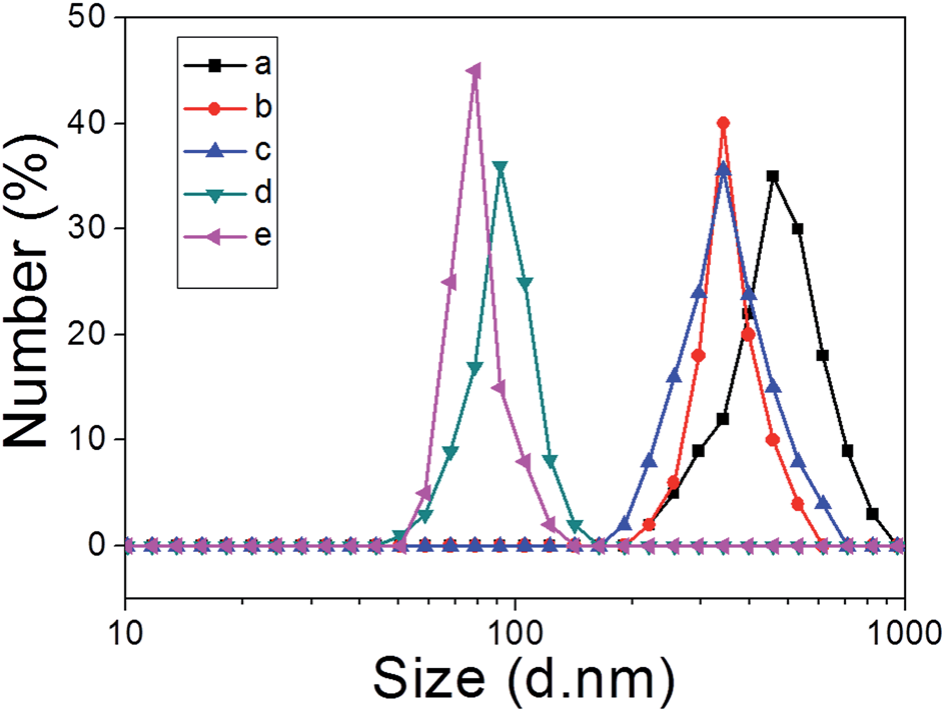
DLS data corresponding to the copolymer aggregates (0.500 mM) produced upon the addition of different amounts of **1** (mM): (a) 0; (b) 0.500; (c) 1.00; (d) 1.50; (e) 2.30 to copolymer **3**.

We next explored whether the self-assembly morphology of the polymer aggregates formed from **3** in water could be modulated *via* the addition of **1**. Transmission electron microscopy (TEM) was used to visualize and track the induced changes. As shown in Fig. 3a, tube-like structures were observed when copolymer **3** (0.100 mM) was added into H_2_O in the absence of any other additive. The maximum axial length of these aggregates was more than several micrometers and their average diameter was about 400 nm, as determined from an analysis of the TEM image. This inferred diameter is in good agreement with the value of 420 nm derived from DLS. Enlarging the TEM image (Fig. 3b) reveals a clear contrast between the grey central part and the dark edge. Such features are characteristic of hollow microtubular structures. Addition of a relatively small amount of **1** (0.500 mM final concentration) to the initial aqueous solution of **3** causes these tubules to vanish completely with the concomitant formation of large vesicular nanostructures (Fig. 3c). The diameters of these vesicles range from 200 to 400 nm and the wall thickness is around 50 nm. When the concentration of **1** is increased to 1.00 mM with that of **3** held constant, evidence of fusion through membrane adhesion is seen; this leads to the formation of strings of beads (Fig. 3d). As the concentration of **1** is further increased to 1.50 mM, these beads constrict themselves resulting in the production of worm-like micelles (Fig. 3e). These latter nanofibers have an average diameter of 80 nm and are several micrometers in length. Finally, when the concentration of **1** is increased to 2.30 mM, the worm-like micelles are replaced by spherical micelles with typical coronacore nanostructures, as inferred from the contrast between the grey periphery and the dark centre seen in the TEM image (Fig. 3f). These spherical micelles are between 50 and 120 nm in diameter. DLS experiments (Fig. 4) were carried out and provided qualitative support for the changes in morphology and aggregate size inferred from the TEM measurements, although slight differences were seen. These differences are ascribed to the fact that the DLS analyses assumed a spherical morphology and thus did not account for the variety of structures seen in Fig. 3.

Taken in concert, the above results provide support for the key design predicate underlying the present study, namely that the nature of the aggregates formed from the amphiphilic copolymer **3** and their evolution can be controlled by varying the relative concentration of macrocycle **1**. This control is ascribed to the macrocycle/anion host–guest recognition between the cation receptor **1** and the carboxylate anion-bearing copolymer **3**.

Based on the observation that the macrocycle/anion complex formed from **1** and **2** is more water soluble than **2** on its own, it is considered likely that the macrocycle/polymer complex involving **1** and **3** will display greater water solubility than the pure copolymer **3**. To the extent that this is true, the copolymer segments bearing **1** as a co-complex will be characterized by enhanced hydrophilicity. In contrast, no change in the hydrophobic nature of the styrene portions is expected. Therefore, increasing the concentration of macrocycle **1** should lead to a gradual increase in the hydrophilic fraction of the macrocycle/copolymer complex, a monotonic variation that will be reflected in macroscopic changes in the self-assembled morphology.^4^,^10^ The presence of greater charge as the result of binding to **1** is also expected to give rise to increased electrostatic repulsion within the hydrophilic fraction of the copolymer. This leads to a higher interfacial free energy. Alterations in micelle shape and curvature allow this increase in free energy to be minimized.^4^,^10^

### *In vitro* cytotoxicity of **1**, **3**, and the aggregates

**Fig. 5:**
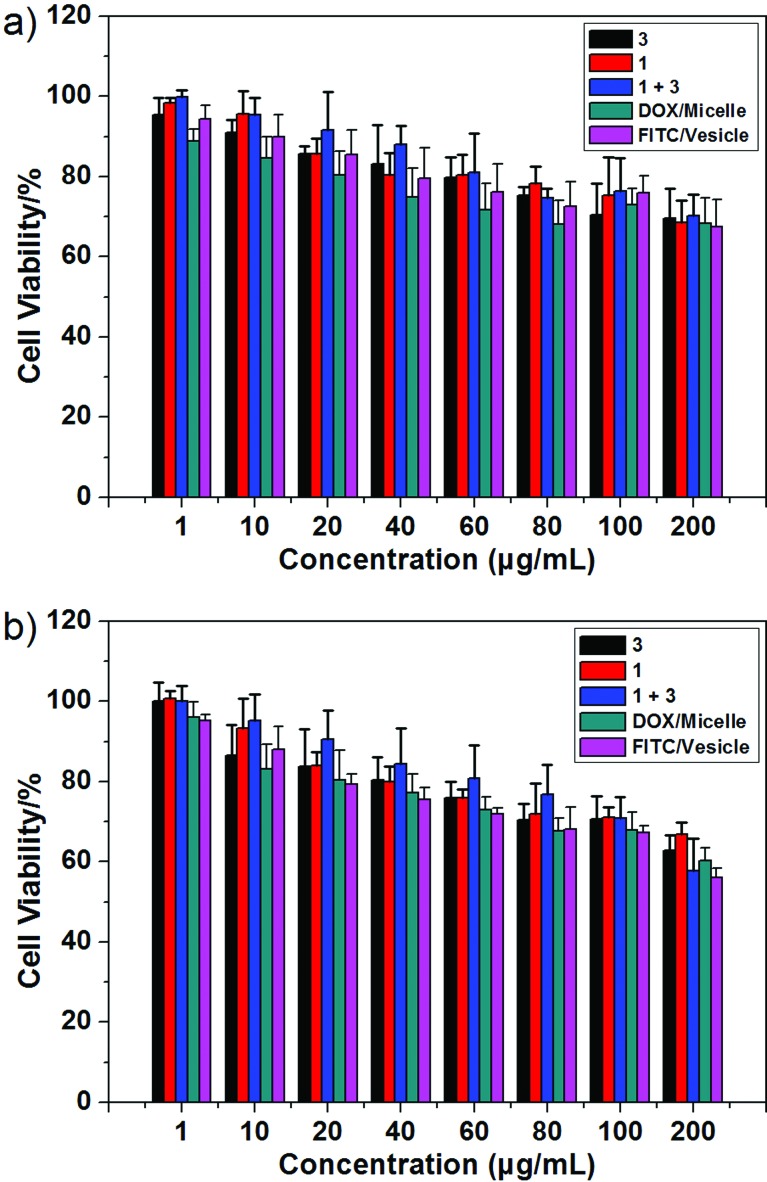
Cytotoxicity of **1**, **3**, the macrocycle/copolymer complex, DOX/micelle, and FITC/vesicle as determined from standard MTT assays using the (a) HUVEC and (b) HepG2 cell lines, respectively.

As a test of whether the present copolymer system might be suitable for use in potential biological applications, the cytotoxicity of **1**, **3**, and the macrocycle/copolymer complex, DOX/micelle, and FITC/vesicle were determined using the HUVEC (human umbilical vein endothelial) and HepG2 (human liver hepatocellular carcinoma) cell lines; this was done using a standard MTT assay.^11^ As can be seen from an inspection of Fig. 5, neither **1**, **3**, the macrocycle/copolymer complex, DOX/micelle, nor FITC/vesicle displayed appreciable cytotoxicity in these cell lines. It was thus inferred that they would display sufficient biocompatibility to warrant further study.

### Cellular internalization of FITC-loaded vesicles and DOX-loaded micelles

**Fig. 6:**
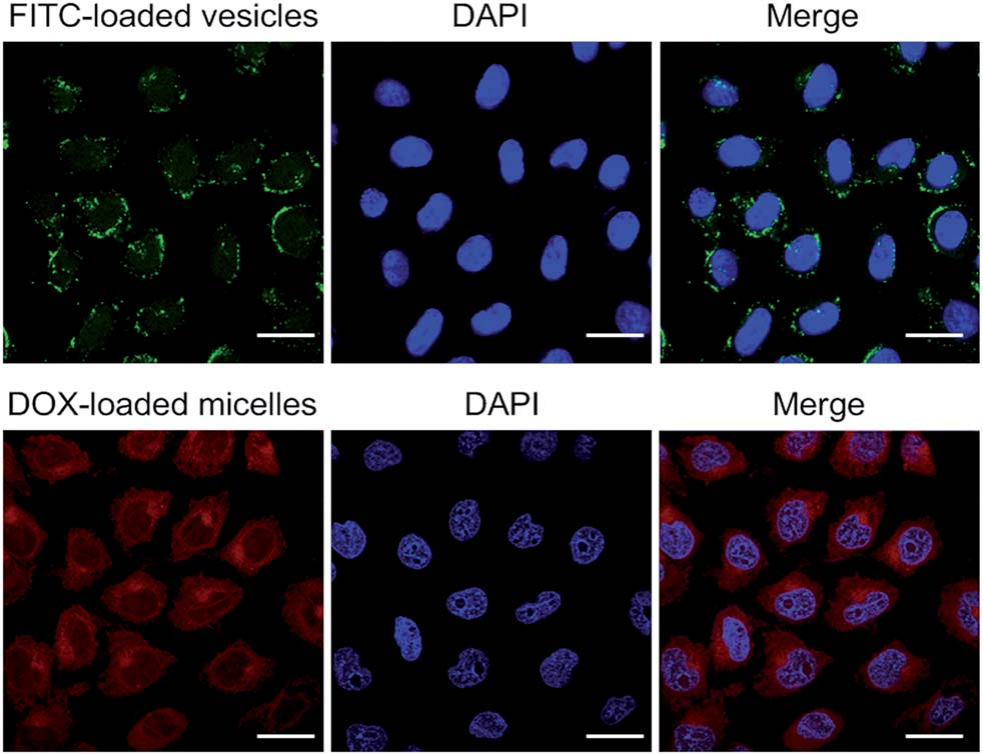
Confocal laser scanning microscopic images of HepG2 cells incubated with FITC-loaded vesicles and DOX-loaded micelles, respectively (scale bar = 20 μm).

The present polymer aggregates were designed to function as rudimentary mimics of natural organelles. As a further test of this analogy, the vesicles and micelles produced from **1** and **3** were used as delivery vehicles. Because the vesicle and micelle forms contain hydrophilic cavities and hydrophobic cores, respectively, it was expected that they could be used in turn to encapsulate hydrophilic and hydrophobic payloads.^11^ The hydrophilic species, fluorescein isothiocyanate (FITC), and the hydrophobic chemotherapeutic, doxorubicin (DOX), were thus used as model payloads. The cellular internalization of the vesicle/FITC and micelle/DOX were examined on HepG2 cells by confocal laser scanning microscopy (CLSM) following the dye-specific fluorescent signature (Fig. 6). After exposure to the vesicle/FITC and micelle/DOX for 4 h, respectively, the vesicle/FITC enriched around the nucleus while the micelle/DOX was internalized into the cells with a fraction of the DOX being delivered into nuclei.^11^ The latter results provide support for the notion that the present system may be used to deliver a chemotherapeutic payload (DOX) into cells. A key goal of the present study was to determine whether this delivery could be controlled by use of a biologically relevant stimulus. Efforts along these lines are discussed below.

### Biological inputs (ATP, ADP, AMP) induced disassembly of the complex formed between **1** and **2**

To date, a number of stimuli-responsive complexes suitable for the delivery of fluorescent chemical entities have been described that respond to *inter alia* chemical or physical stimuli,^14^ including voltage changes,14*a* light,14*b* pH differences,14c–e exposure to gases,14*f* redox perturbations,14*g**etc.* As would be expected given the mode of interaction, the control complex formed between **1** and **2** proved pH responsive (ESI, Fig. S15 and S16†). Such pH-based switching effects could also be used to control the aggregates between **1** and **3** (ESI, Fig. S17–S19†). However, for use in biological applications, a more active signaling approach was sought, in part because it might allow in due course a level of control that could be used to prevent excess accumulation of chemical agent under conditions of, *e.g.*, chemotherapeutic adminstration.^12^ As a proof-of-principle test of this postulate studies were carried out using ATP, ADP, and AMP as potential signaling agents.

**Fig. 7:**
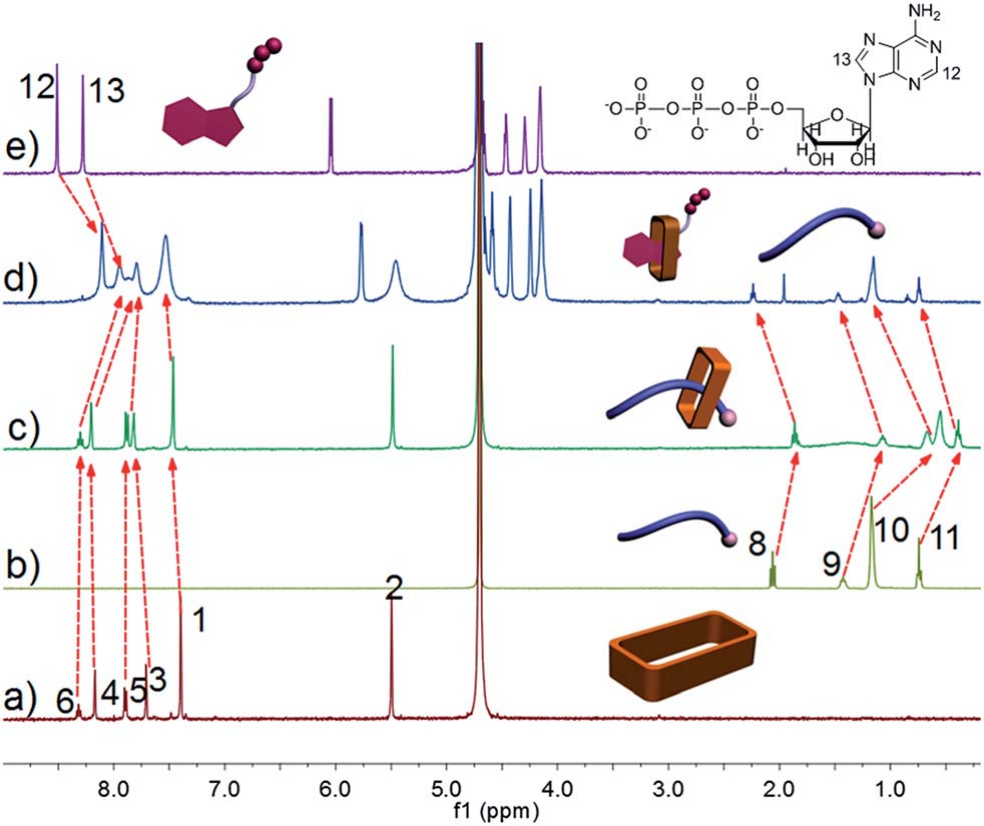
^1^H NMR spectra (500 MHz, D_2_O, 298 K): (a) 3.00 mM **1**; (b) 3.00 mM **2**; (c) **1** and **2** (3.00 mM for each); (c) **1**, **2**, and ATP (3.00 mM for each); (e) 3.00 mM ATP.

ATP is a critical biological signaling molecule and near-ubiquitous source of cellular energy. Due to the presence of three anionic phosphate groups in ATP under physiological conditions, we consider it likely that ATP would serve as a competitive guest capable of triggering the disassembly of the complex formed between **1** and **2**. As can be seen from an inspection of Fig. 7, after mixing **1** and **2**, all the proton signals (H_8_–H_11_) of **2** undergo upfield shifts (spectra b and c). On the other hand, the addition of ATP caused the chemical shift of these signals to revert back essentially to those of the uncomplexed form (spectra b and d). In addition, the signals corresponding to protons H_12_ and H_13_ of ATP underwent an upfield shift. These findings are consistent with the interaction between **1** and **2** being essentially reversed and ATP being bound by **1**. On this basis, it was expected that the aggregates formed *via* the self-assembly of **3** and **1** could likewise be destroyed by adding ATP.

Since ADP (two anionic phosphate groups) and AMP (one anionic phosphate group) also possess negative charges, their ability to compete with guest **2** was also examined (ESI. Fig. S20 and S21†). After adding ADP or AMP to a mixture of **1** and **2**, the signals corresponding to protons present in **2** moved back towards the values for the uncomplexed form, while the resonances for protons H_14_, H_15_ and H_16_, H_17_ of ADP and AMP, respectively, shifted to higher field. However, the extent of the effect was less than that in the case of ATP. For example, after mixing **1** and **2**, the chemical shift of proton H_10_ in **2** changed from 1.17 ppm to 0.536 and 0.675 ppm, respectively. In the presence of equal concentrations of ATP, ADP, and AMP, this diagnostic resonance was shifted 1.15, 1.05, and 0.981 ppm in the case of these three separate experiments, respectively. We thus conclude that among this set of nucleotides, ATP would be the most efficient trigger for inducing a change in copolymer morphology and affecting release of an appropriately chosen payload.

### Biological signals (ATP, ADP, AMP) induced drug release

**Fig. 8:**
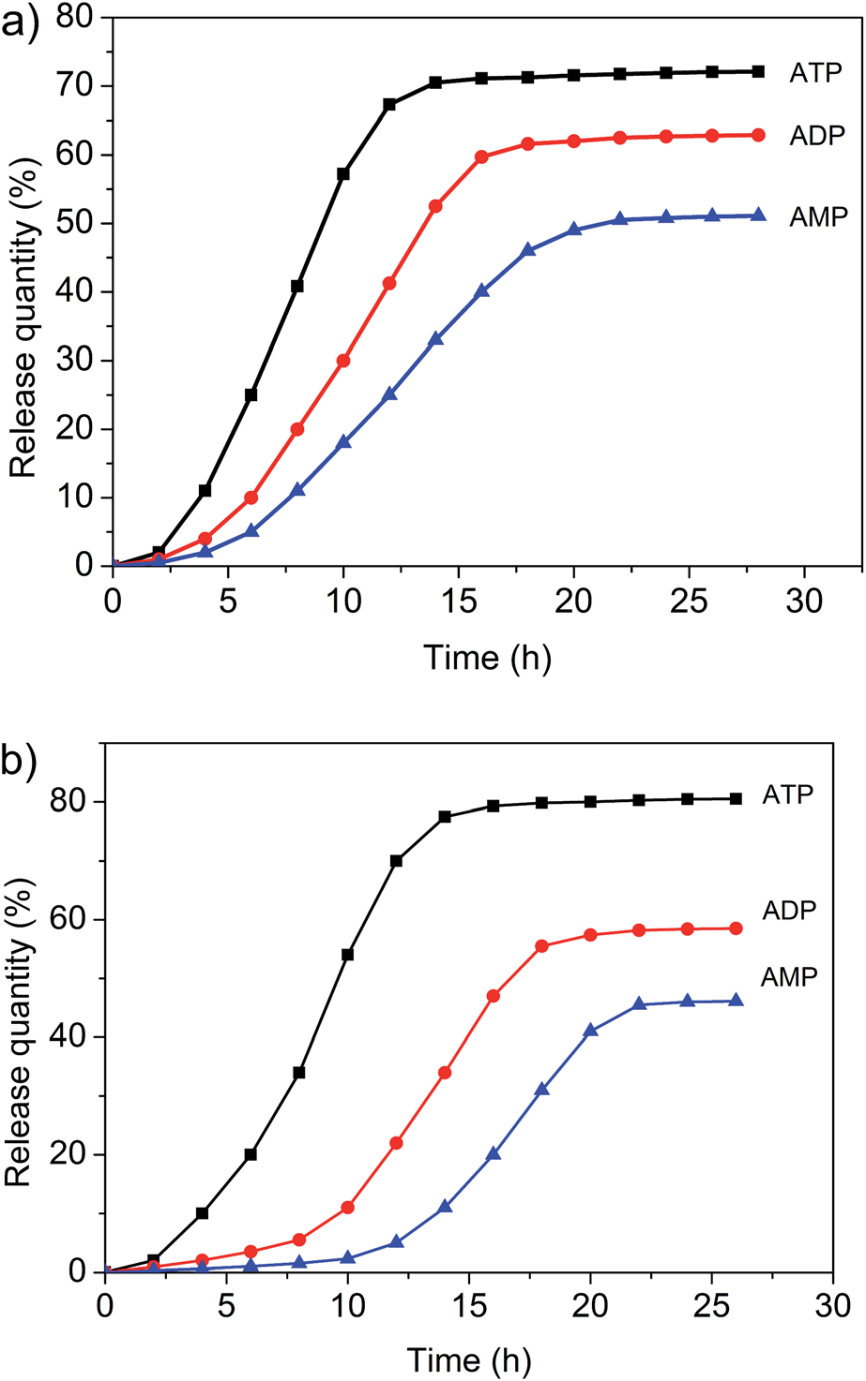
(a) Controlled drug release of DOX from the self-assembled polymeric micelles formed from **3** (0.100 mM) and **1** (0.500 mM) upon the addition of different intracellular biological signaling agents (pH = 7.0): ATP (2.30 mM), ADP (2.30 mM), and AMP (2.30 mM). (b) Controlled drug release of FITC from the polymer vesicles (0.100 mM **3** and 2.30 mM **1**) upon the addition of different intracellular biological signaling agents (pH = 7.0): ATP (2.30 mM), ADP (2.30 mM), and AMP (2.30 mM).

Both the micelles and vesicles formed from **1** and **3** were expected to undergo controlled disaggregation in the presence of ATP, ADP, and AMP since they should compete with the key host–guest anion binding interactions that stabilize these structures (Fig. 7, ESI. Fig. S20 and S21†). To the extent that this supposition is correct, the addition of these nucleotides to the aggregates used to encapsulate FITC and DOX would serve to promote release. FITC and DOX differ in their optical properties. As noted above, the fluorescence emission intensity of DOX is higher in hydrophobic environments, whereas that of FITC is higher in aqueous media.^3^,^6^,^11^ Addition of ATP, ADP, and AMP to the micelle/DOX solution lead to a decrease in the fluorescence intensity of the sample in both a time and nucleotide-dependent manner (ESI. Fig. S22–S24†). As might be expected, at any given time point, ATP was the most efficient trigger (Fig. 8). Such findings are consistent with the suggestion that the relative concentration of DOX within the hydrophobic micellular environment is reduced as the result of its release into the bulk aqueous medium. In contrast, the fluorescence intensity of the vesicle/FITC sample increased upon the addition of these biological triggers (Fig. S25–S27†), as would be expected under conditions where the bound FITC is released. Again nucleotide dependent behaviour was seen with ATP being the most effective trigger at any given comparable time point (Fig. 8b). These results are thus taken as support for the notion that the diversiform structures made up from **1** and **3** may prove useful as small molecule carriers and transport agents.

## Conclusions

In summary, we report the preparation of a water-soluble form of the ‘Texas-sized’ molecular box **1** and basic studies of its macrocycle/anion host–guest chemistry in water. Upon the addition of box **1** to an amphiphilic copolymer bearing carboxylate anion functionality, changes in the nature of the self-assembled aggregates are observed that are ascribed to changes in the hydrophilic–hydrophobic ratio within the copolymer. The present system thus serves as a synthetic model for the morphological changes seen in natural organelles and living cells. It also shows promise as a biological signal-responsive delivery system capable of binding and releasing very different payloads, including an established cancer chemotherapeutic agent (DOX) and a widely utilized fluorophore (FITC), based on the specific diversiform used. The elevated levels of ATP found in cancer cells leads us to predict that constructs such as those reported here that are able to transport chosen payloads and then release them selectively in the presence of ATP might have a role to play in the tumour-targeted delivery of diagnostic and therapeutic agents. Current work is designed to explore potential applications of this and related systems in the life science, biomedical, and photoelectric materials areas.

## Conflict of interest

The authors declare no competing financial interest.

## Supplementary Material

Supplementary information
