# Supplementary material for: Controlling amphiphilic copolymer self-assembly morphologies based on macrocycle/anion recognition and nucleotide-induced payload release
Source: Chem Sci. 2016 May 24;7(9):6006–14. doi: 10.1039/c6sc01851c (PMC5015656; doi:10.1039/c6sc01851c)
Supplement: Supplementary file 1 [file SC-007-C6SC01851C-s001.pdf]

Electronic Supplementary Material (ESI) for Chemical Science.
This journal is © The Royal Society of Chemistry 2016

Controlling amphiphilic copolymer self-assembly morphologies based on macrocycle/anion recognition and nucleotide-induced payload release

Xiaofan Ji,‡[a] Hu Wang,‡[a] Yang Li,[a] Danyu Xia,[a] Hao Li,[b,a] Guping Tang,[a] Jonathan L. Sessler,*[,b,c] and Feihe Huang*[,a]

[a] *State Key Laboratory of Chemical Engineering, Center for Chemistry of High-Performance & Novel Materials, Department of Chemistry, Zhejiang University, Hangzhou 310027, P. R. China; Fax and Tel: +86-571-8795-3189; Email: fhuang@zju.edu.cn*

[b] *Department of Chemistry, 105 East 24th Street, Stop A5300, The University of Texas at Austin, Austin, Texas 78712, United States; Email: sessler@cm.utexas.edu*

[c] *Institute for Supramolecular and Catalytic Chemistry, Shanghai University, Shanghai 200444, China*

‡ These authors contributed equally to this work.

Electronic Supplementary Information

1. *Materials and methods* S3
2. *Characterization data for **1*** S4
3. *Full view of the NOSY NMR spectrum of a mixture of **1** and **2*** S8
4. *Stoichiometry and association constant determination for the complex formed between **1** and **2*** S9
5. *Characterization of **5** and **3*** S12
6. *pH-responsiveness of the host−guest interactions* S15
7. *ADP and AMP induced NMR spectrum changes* S18
8. *Nucleotide-induced release experiments* S20
9. *The stability of different aggregates* S23
10. *A TEM image with two types of aggregates* S23
11. *TEM images after loading DOX or FITC* S23
12. *DLS after loading DOX or FITC* S24
13. *The drug loading percentages* S24
14. *References* S24

1. Materials and methods

Compounds **4**[S1] and **7**[S2] were prepared according to literature procedures. Solvents were either employed as purchased or dried according to procedures described in the literature. ^{1}H NMR spectra were collected on a Bruker Advance DMX-500 spectrometer using TMS as an internal standard. Low-resolution electrospray ionization mass spectra (LRESI-MS) were obtained on a Bruker Esquire 3000 plus mass spectrometer (Bruker-Franzen Analytik GmbH Bremen, Germany) equipped with an ESI interface and an ion trap analyzer. High-resolution electrospray ionization mass spectra (HRESI-MS) were obtained on a Bruker 7-Tesla FT-ICR mass spectrometer equipped with an electrospray source (Billerica, MA, USA). Molecular weight distributions were measured on a conventional gel permeation chromatography (GPC) system equipped with a Waters 1525 Isocratic HPLC pump, a Waters 2414 refractive index detector, and a set of Waters Styragel columns (HR1, HR2 and HR4, 7.8 mm × 300 mm). GPC measurements were carried out at 35 °C using THF as the solvent with a flow rate of 1.0 mL/min. The system was calibrated with linear polystyrene standards. Dynamic light scattering (DLS) was carried out on a Malvern Nanosizer S instrument at room temperature. Transmission electron microscopy investigations were carried out on a HITACHI HT-7700 instrument. The fluorescence spectra were recorded on a Perkin Elmer LS55 fluorescence spectrophotometer.

*2. Characterization data for **1***

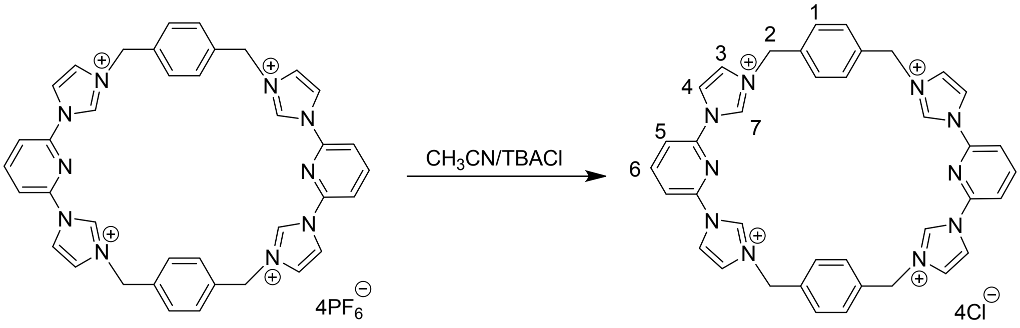

4 **1**

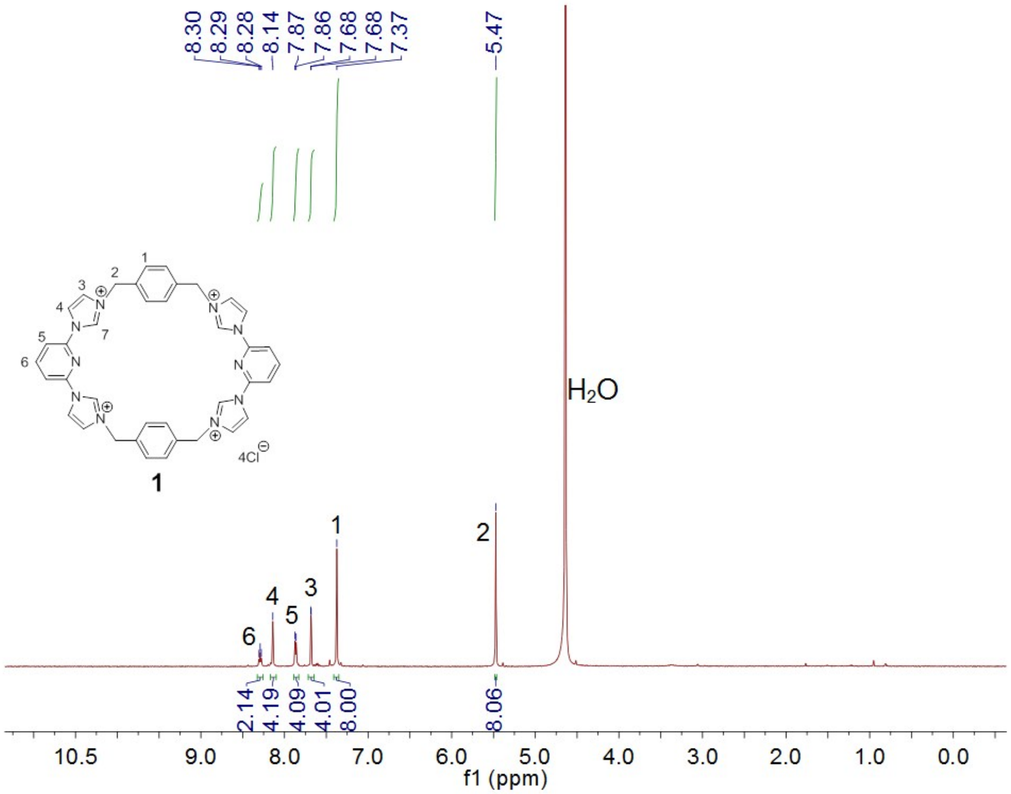

Fig. S1. ^{1}H NMR spectrum (500 MHz, D_2O, 298 K) of **1**.

The relatively more acidic proton H_7 on the imidazole moiety present in **1** undergoes exchange with the deuterium atoms of D_2O. As a consequence no signal for this proton is observed in the proton NMR spectrum.

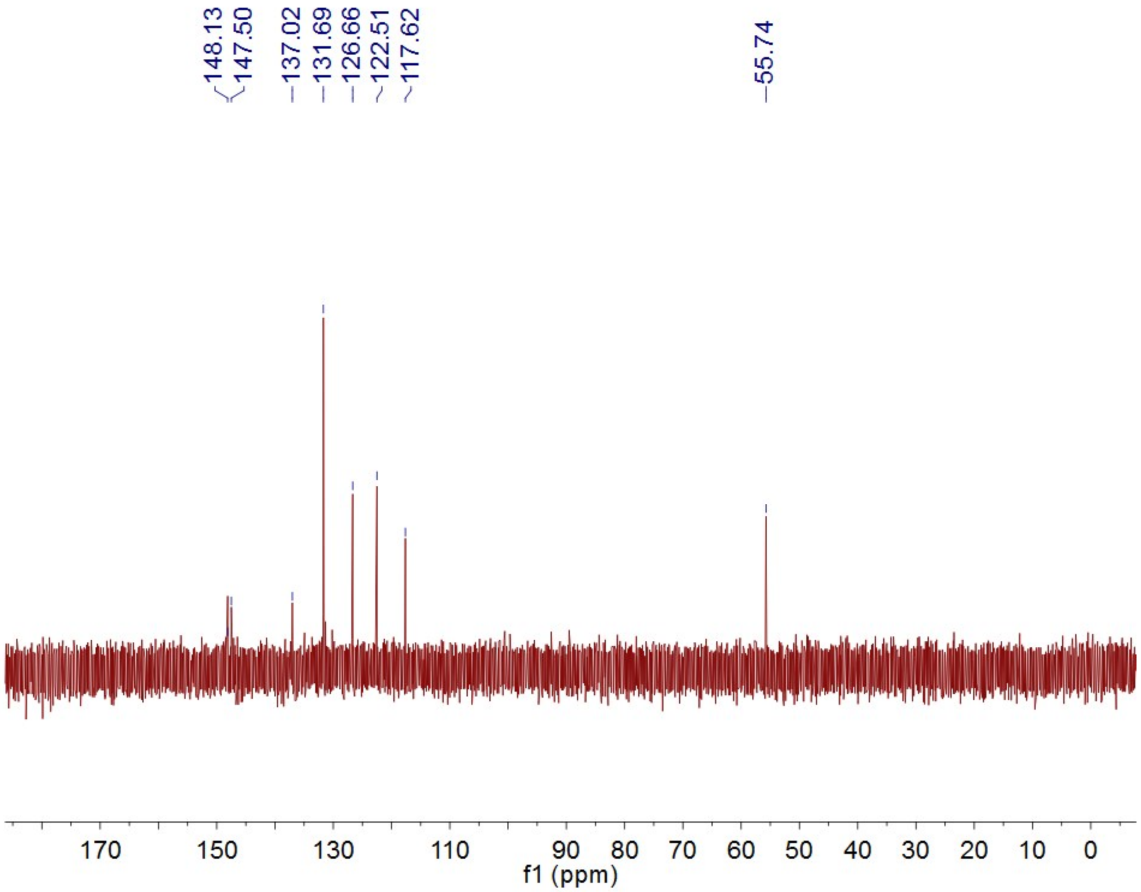

Fig. S2. ^{13}C NMR spectrum (500 MHz, D_2O, 298 K) of **1**.

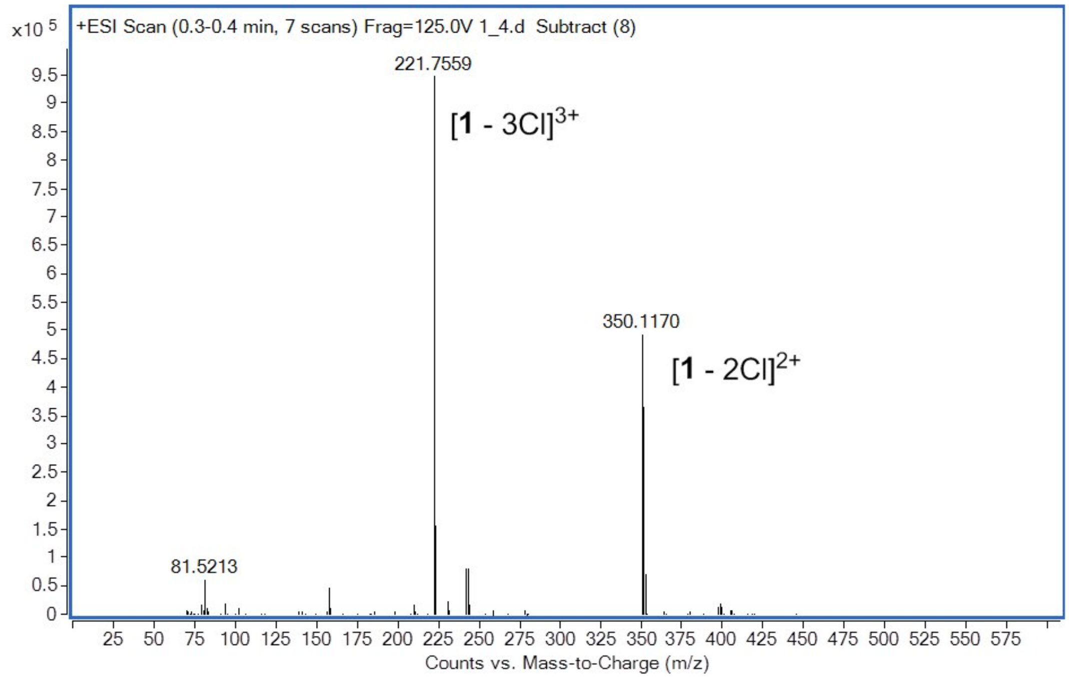

Fig. S3. LR ESI mass spectrum of **1**.

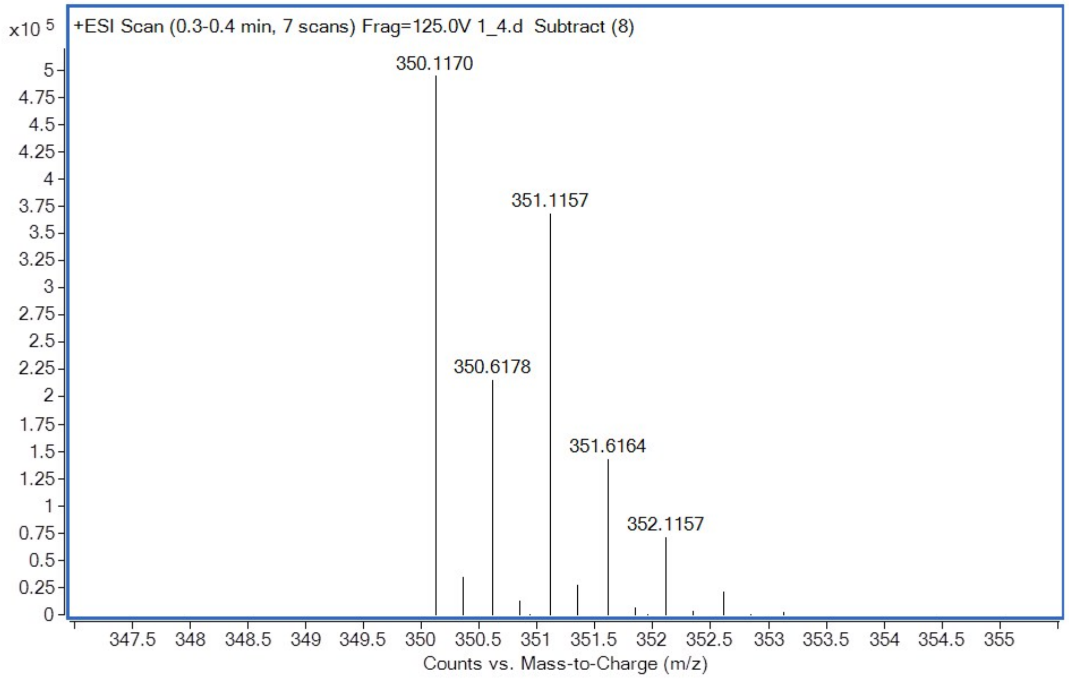

Fig. S4. HR ESI mass spectrum of **1**.

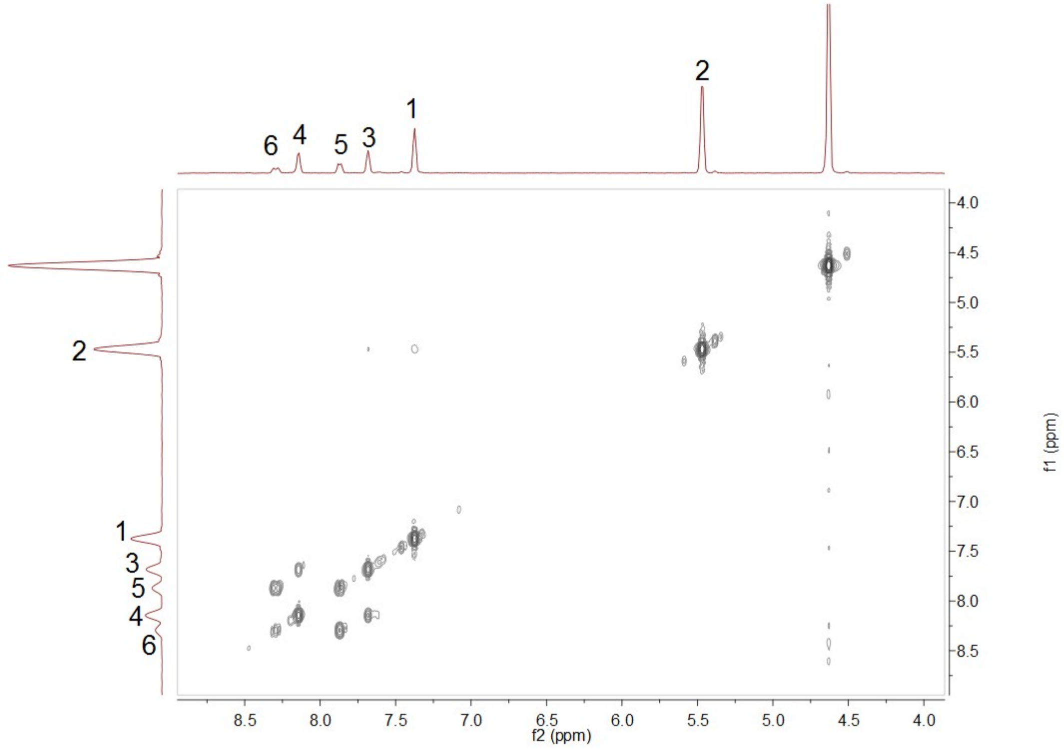

Fig. S5. Full view of the COSY NMR spectrum (500 MHz, D_2O, 298 K, 1.00 mM) of **1**.

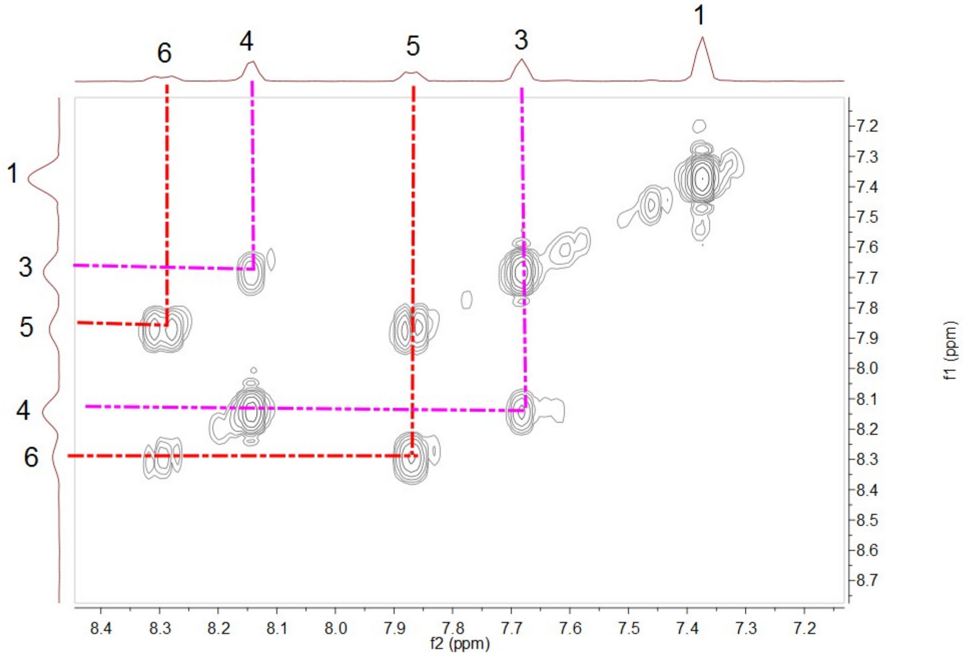

Fig. S6. Expanded view of the COSY NMR spectrum (500 MHz, D_2O, 298 K, 1.00 mM) of **1**.

Correlated signals were observed between H_3 and H_4 and between H_5 and H_6 in D_2O.

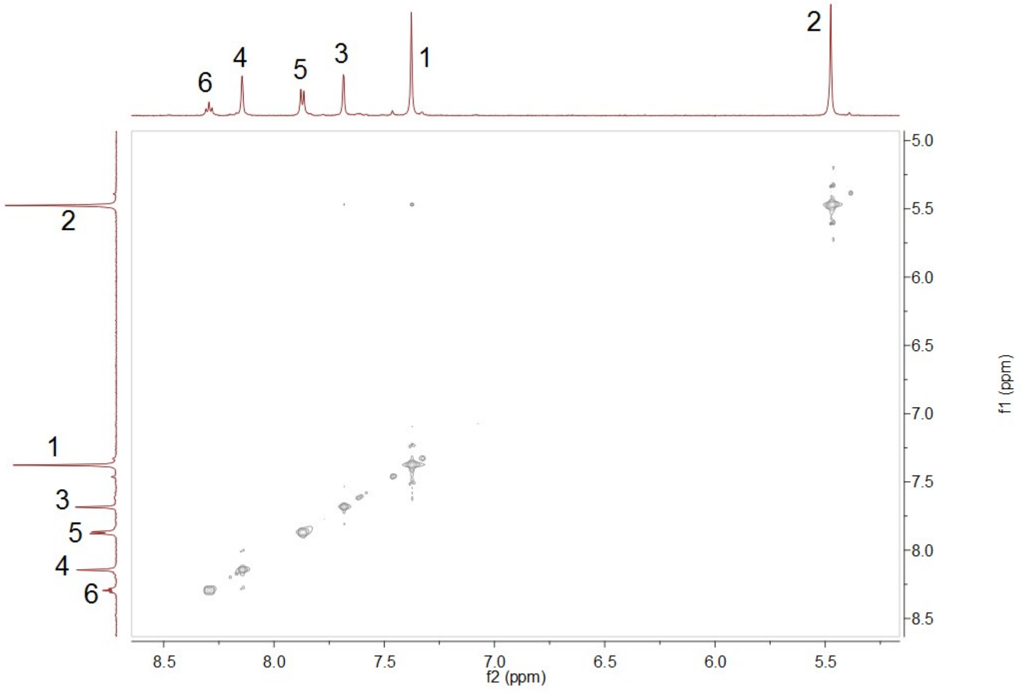

Fig. S7. NOSY NMR spectrum (500 MHz, D_2O, 298 K, 1.00 mM) of **1**.

*3. Full view of the NOSY NMR spectrum of a mixture of **1** and **2***

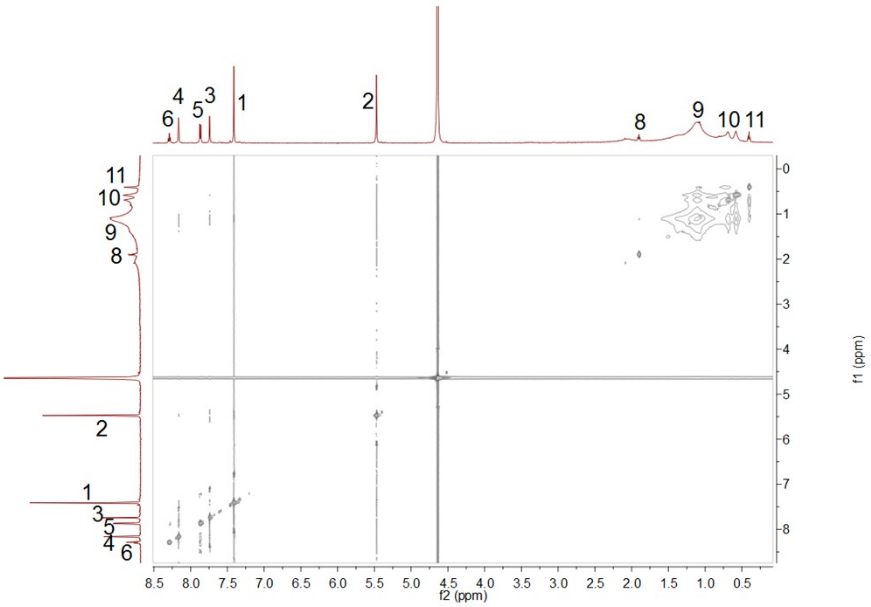

Fig. S8. Full view of the NOSY NMR spectrum (600 MHz, D_2O, 298 K) of **1** and **2** (3.00 mM for each).

*4. Stoichiometry and association constant determination for the complex formed between **1** and **2***

To determine the stoichiometry and association constant corresponding to the interaction between macrocycle **1** and ammonium decanoate **2**, ^{1}H NMR titrations were carried out using solutions that had a constant concentration of **1** (0.500 mM) and varying concentrations of **2**. Using a non-linear curve-fitting method, the association constant between guest **2** and receptor **1** was calculated. From a mole ratio plot, evidence for a 1:1 stoichiometry was obtained.

The non-linear curve-fitting was based on the equation:[S3]

$\Delta\delta = (\Delta\delta_\infty/[G]_0)\ (0.5[H]_0 + 0.5([G]_0+1/K_a)-(0.5\ ([H]_0{}^2+(2[H]_0(1/K_a - [G]_0)) + (1/K_a + [G]_0)^2)\ ^{0.5}))$ (Eq. S1)

Where $\Delta\delta$ is the chemical shift change of H_3 on **1** at $[G]_0$, $\Delta\delta_\infty$ is the chemical shift change of H_3 when the host is completely complexed, $[H]_0$ is the fixed initial concentration of the host **1**, and $[G]_0$ is the varying concentration of the guest **2**.

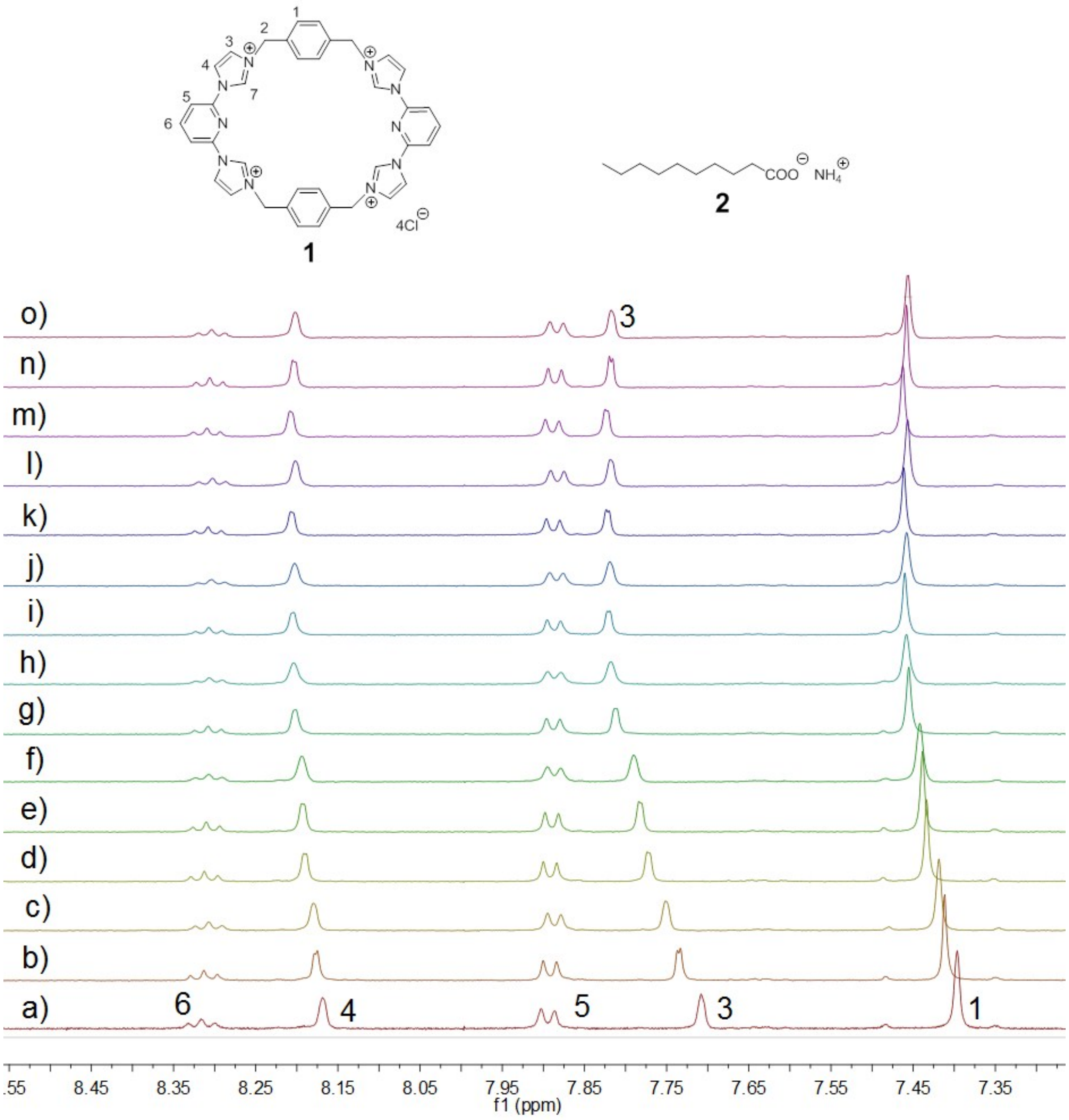

Fig. S9. ^{1}H NMR spectra (D_2O, 293 K, 500 MHz) of **1** recorded at a concentration of 0.500 mM in the presence of differing concentrations of **2**: (a) 0.00 mM; (b) 0.113 mM; (c) 0.214 mM; (d) 0.305 mM; (e) 0.387 mM; (f) 0.461 mM; (g) 0.667 mM; (h) 0.824 mM; (i) 0.947 mM; (j) 1.05 mM; (k) 1.13 mM; (l) 1.20 mM; (m) 1.31 mM; (n) 1.39 mM; (o) 1.46 mM.

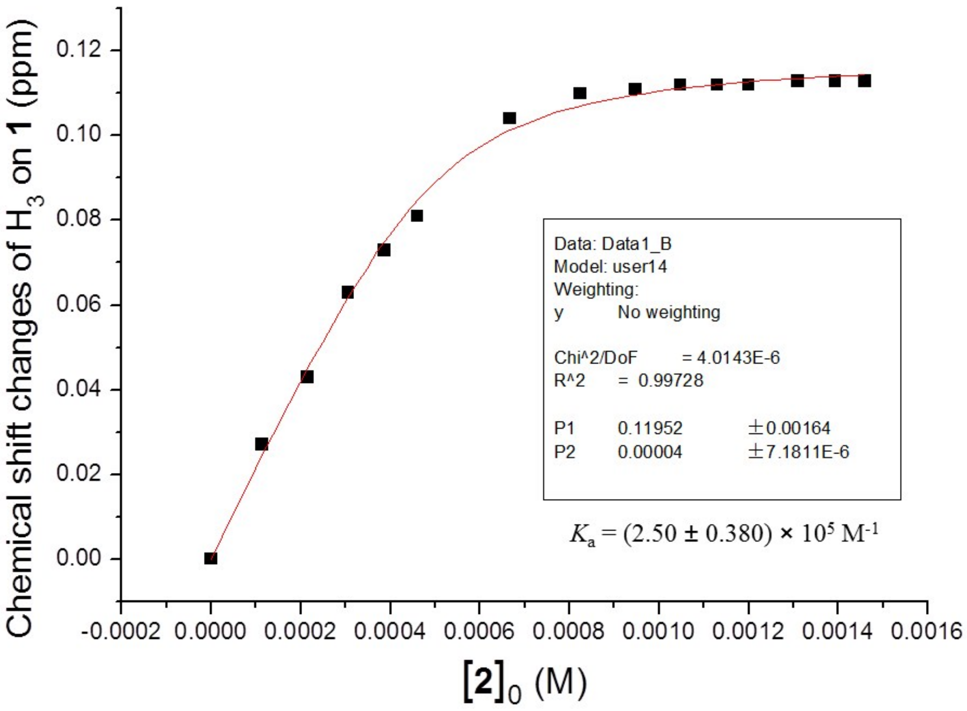

Fig. S10. Changes in the chemical shift corresponding to H_3 on **1** as a function of added **2**. The black squares are data points observed by experiments, whereas the red solid line was obtained from the non-linear curve-fitting using Eq. S1.

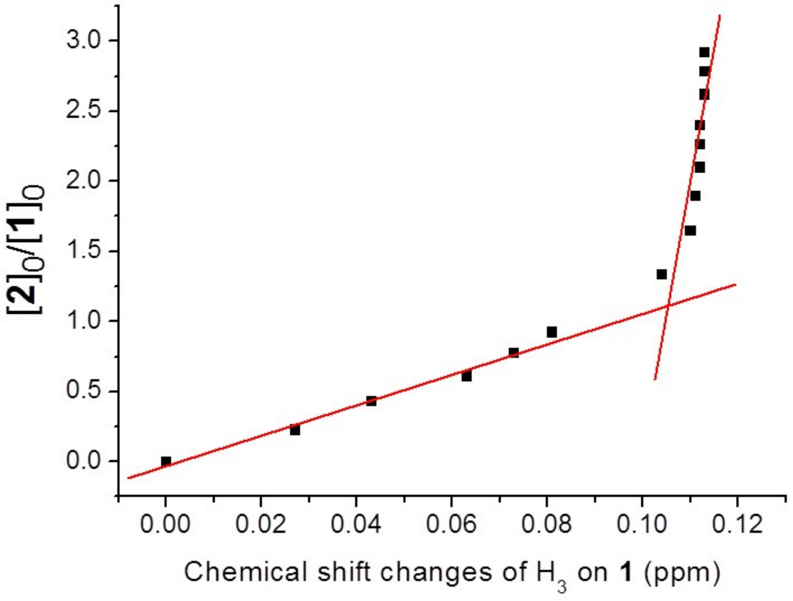

Fig. S11. Mole ratio plot for the interaction between **1** and **2**; the break seen at a 1:1 molar ratio of these two species was taken as an indication of a 1:1 binding stoichiometry.

*5. Characterization of **5** and **3***

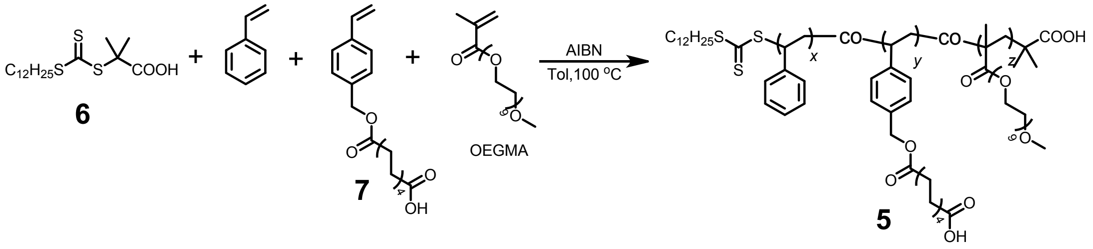

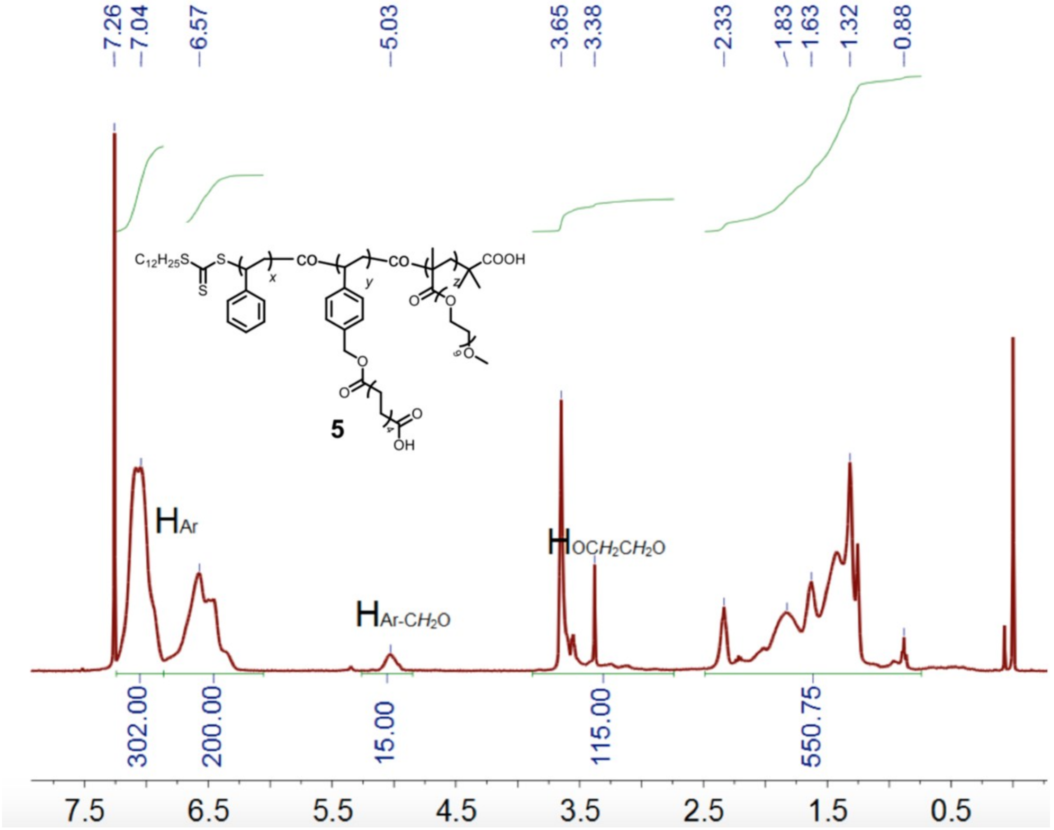

Fig. S12. ^{1}H NMR spectrum (500 MHz, $CDCl_3$, 298 K) of **5**.

The ratio of *x*/*y*/*z* is 54.4/4.4/1.7, as calculated from integrations of the protons present in the *Ar*, Ar-CH_2O, and OCH_2CH_2O subunits, respectively. From the M_n value and this *x*/*y*/*z* ratio, the values of *x*, *y*, and *z* were calculated to be 285, 23 and 9, respectively.

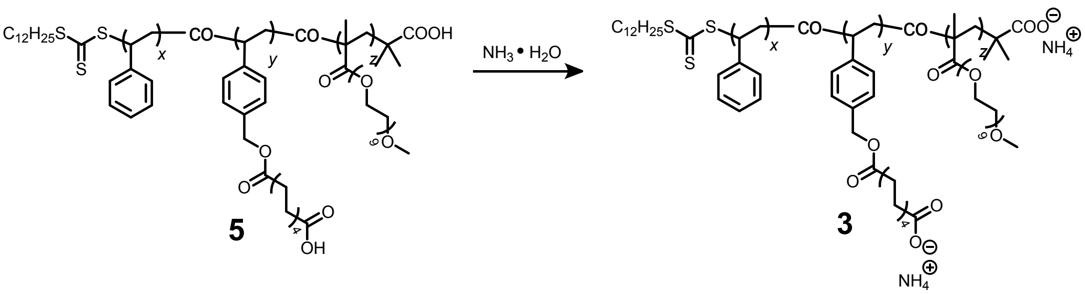

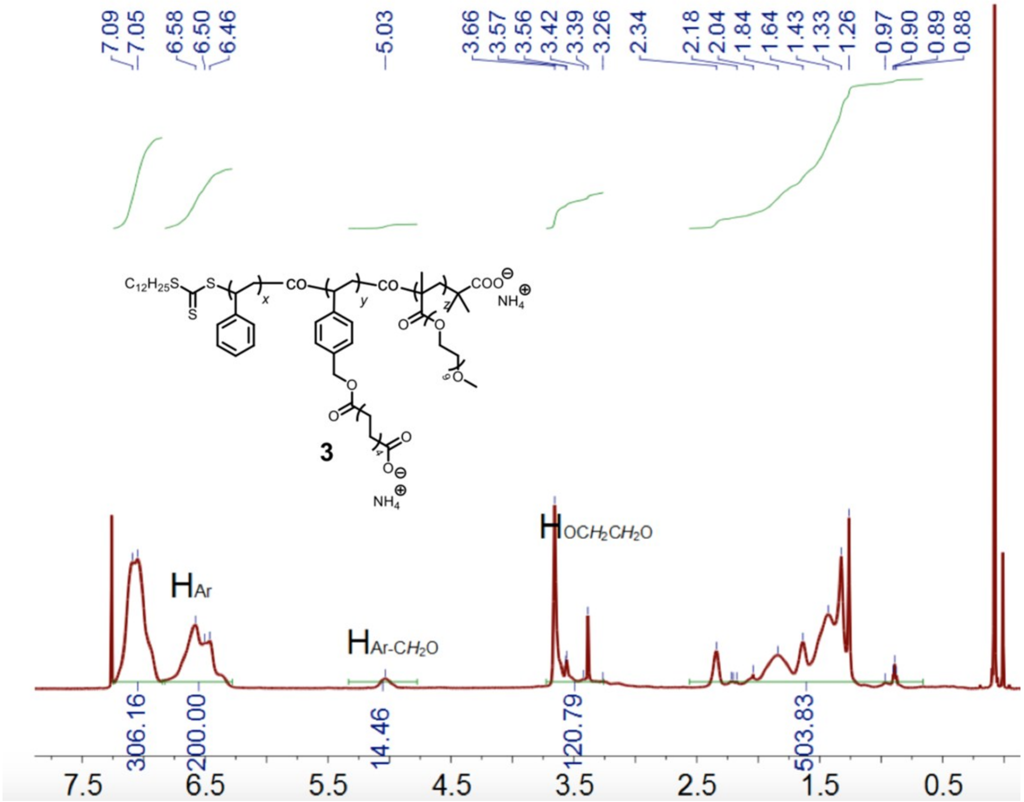

Fig. S13. ^{1}H NMR spectrum (500 MHz, $CDCl_3$, 298 K) of **3**.

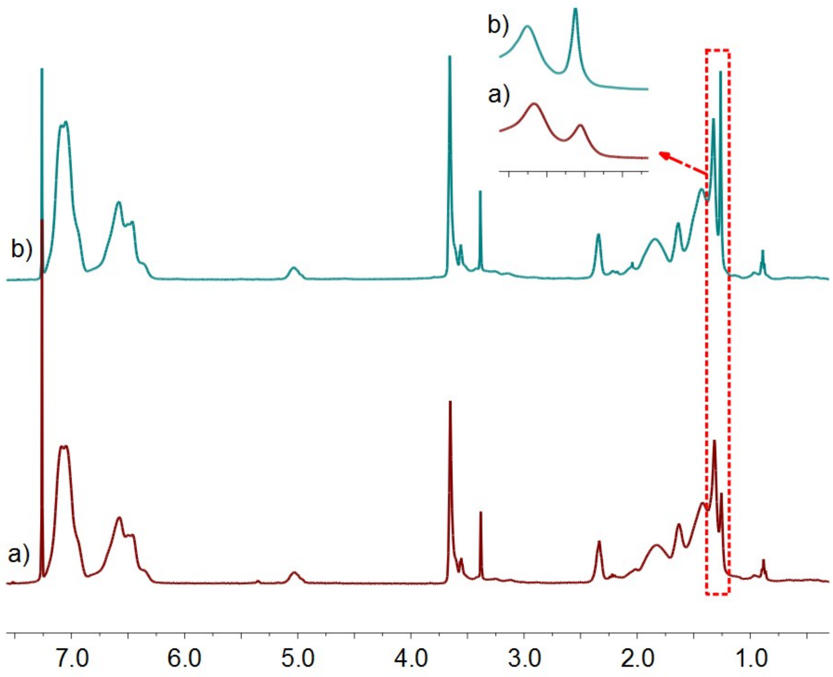

Fig. S14. Partial ^{1}H NMR spectra (500 MHz, $CDCl_3$, 293 K): (a) **5** (1.71×10^{-5} M); (b) **3** (1.71×10^{-5} M); insert: enlarged picture of peaks from 1.4 to 1.2 ppm. It can be seen from an inspection of this fig. that the peak corresponding to the alkyl chain protons undergoes a change consistent with the conversion of the neutral polymer **5** into its corresponding salt form **3**.

6. pH-responsiveness of the host–guest interactions

It is well known that the anionic carboxylate group can be converted into the neutral carboxylic group by decreasing the solution pH; thus the carboxylate group of **2** can be changed to their corresponding acid form by adding aqueous HCl to the initial aqueous medium. This leads to disassembly of complex **1**⊃**2**. As shown in Figs. S15 and S16, when an aqueous HCl solution was added to a solution of **1** and **2** in D_2O, the chemical shift values corresponding to protons present in **1** and **2** revert essentially to those of their uncomplexed forms, a result consistent with the interaction between **1** and **2** being essentially reversed. On this basis, we felt it likely that the supramolecular construct made up from **1** and **3** would also show pH-dependent behavior. Although not a point of emphasis is this submission, support for this contention has been obtained and is given below.

The vesicles and micelles produced from **1** and **3** were used as delivery vehicles. Both sets of structures were expected to be destroyed by decreasing the pH, since this would compete with the key host–guest anion binding interaction. Because the vesicle and micelle forms contain hydrophilic cavities and hydrophobic cores, respectively, it was expected that they could be used in turn to encapsulate hydrophilic and hydrophobic cargoes. The hydrophilic species, fluorescein isothiocyanate (FITC), and the hydrophobic chemo-therapeutic, doxorubicin (DOX), were thus used as model cargoes. These two species differ in their optical properties. As confirmed by NMR spectroscopic analyses (Figs. S15 and S16), adding acid to the aggregates used to encapsulate FITC and DOX serves to induce release (Figs. S17–S19). 1.0 mL of the micelle/DOX sample was taken out, and the amount of released FITC at different pH values was determined *via* fluorescence measurements. As shown in Fig. S17c, as the pH of the micelle/DOX solution is lowered, the fluorescence intensity of the sample decreased. Such a finding is consistent with the relative concentration of DOX within the hydrophobic micellular environment being reduced as the result of its release into the bulk aqueous medium. Similarly, 1.0 mL of the vesicle/FITC sample was also taken out, and the amount of released FITC at different pH values was determined *via* fluorescence measurements. In contrast, the fluorescence intensity of the vesicle/FITC sample increased as the pH was lowered (Fig. S17d), as would be expected under conditions where the bound FITC is released. Moreover, TEM images of the final materials were consistent with the micelle (Fig. S18) and vesicle (Fig. S19) structures having been destroyed, respectively.

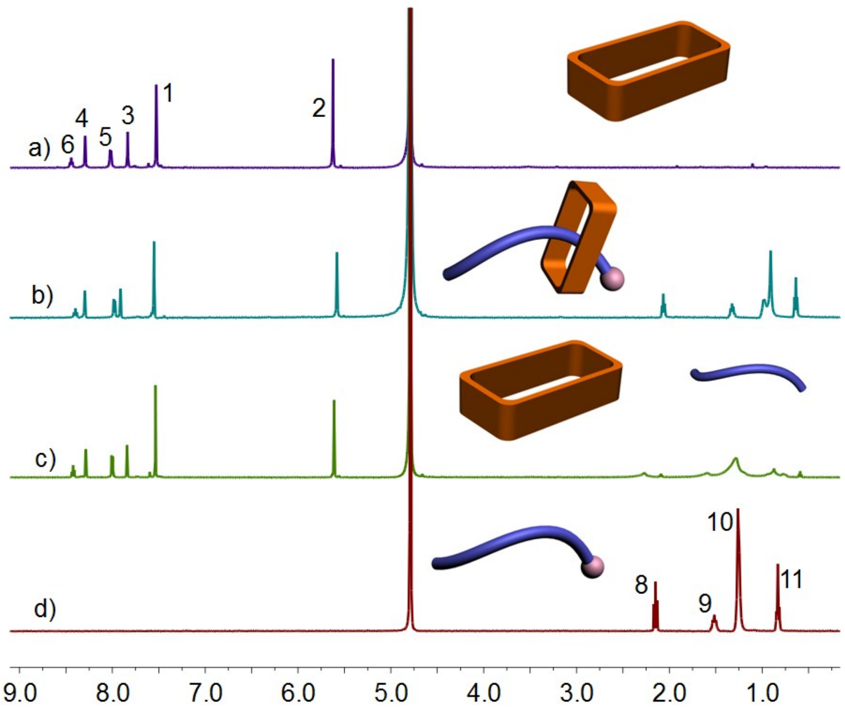

Fig. S15. ^{1}H NMR spectra (500 MHz, D_2O, 298 K): a) 3.00 mM **1**; b) 3.00 mM **2** and 3.00 mM **3**; c) 4.00 mM HCl, 3.00 mM **1**, and 3.00 mM **2**; d) 3.00 mM **2**.

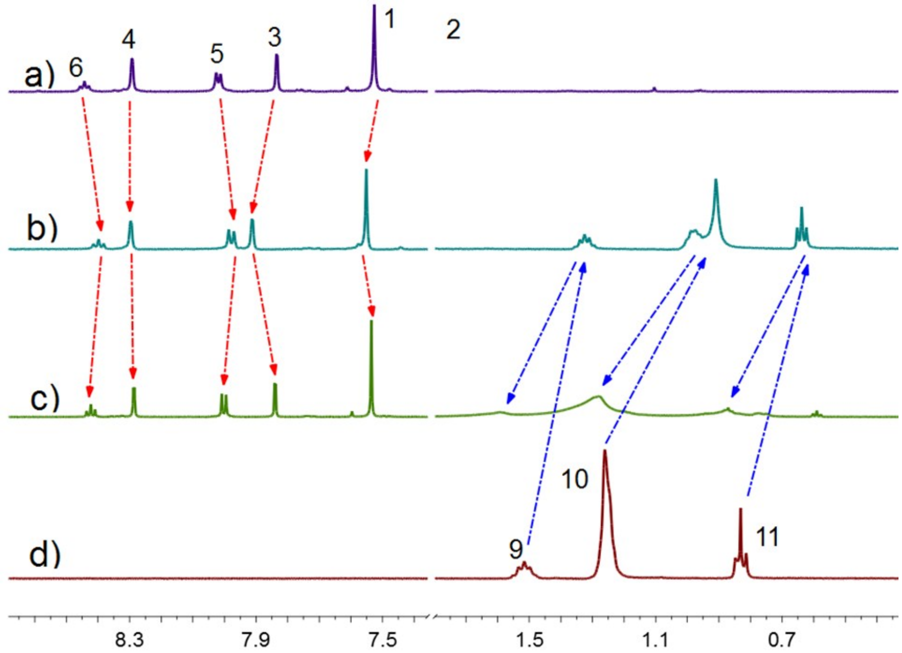

Fig. S16. Partial ^{1}H NMR spectra representing an expanded view of the spectra shown in Fig. S15.

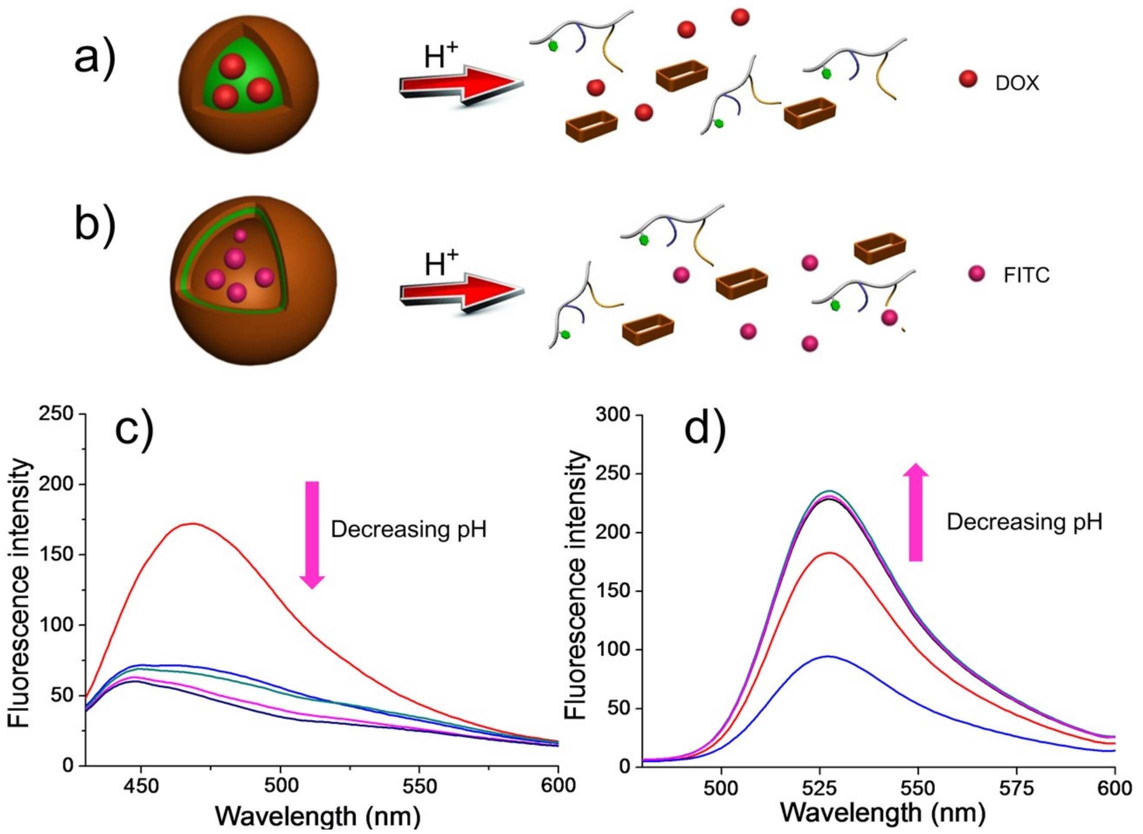

Fig. S17. Cartoon representation of the pH-controlled release of DOX (a) and FITC (b); (c) fluorescence spectra of the micelle/DOX solution recorded at different pH values. From top to bottom: 7.0, 6.0, 5.5, 5.0, 4.5; (d) fluorescence spectra of the vesicle/FITC solution recorded at different pH values. From bottom to top: 7.0, 6.0, 5.5, 5.0, 4.5.

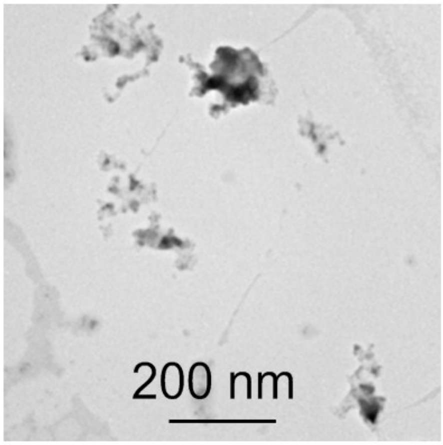

Fig. S18. TEM image of the micelle/DOX material produced at pH = 4.5.

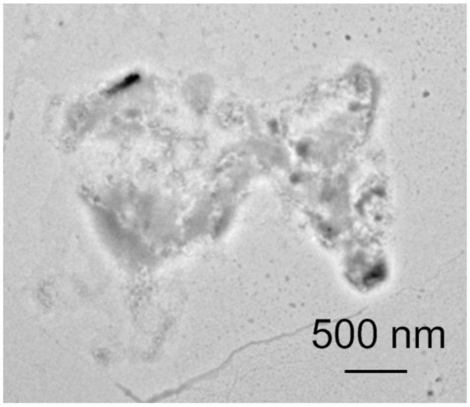

Fig. S19. TEM image of the vesicle/FITC product obtained from a pH = 4.5 solution.

7. ADP and AMP induced NMR spectrum changes

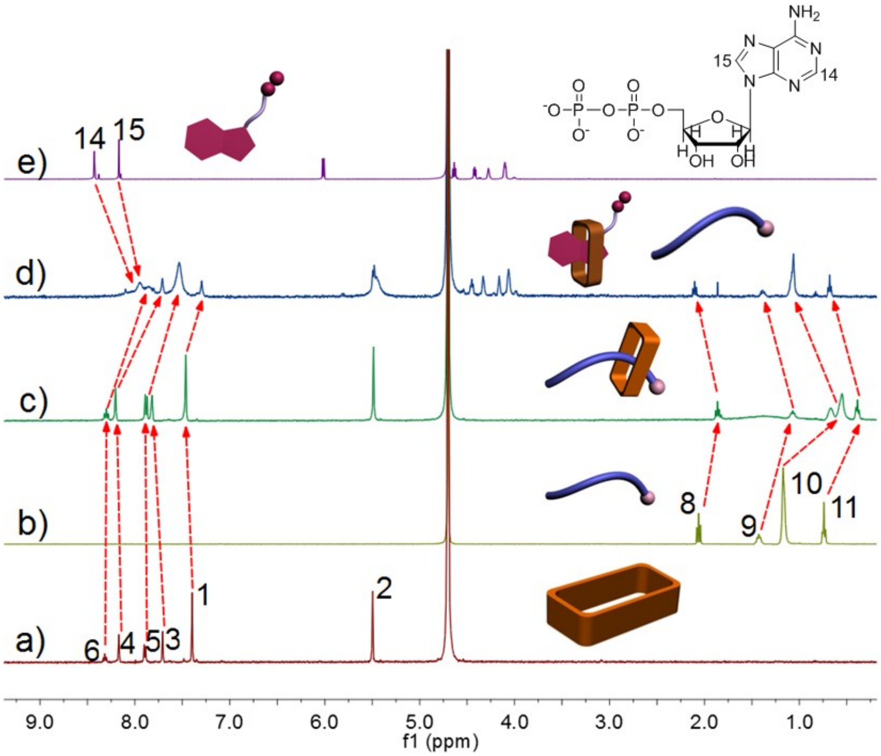

Fig. S20. ^1H NMR spectra (500 MHz, D_2O, 298 K): a) 3.00 mM **1**; b) 3.00 mM **2**; c) **1** and **2** (3.00 mM for each); c) **1**, **2**, and ADP (3.00 mM for each); e) 3.00 mM ADP.

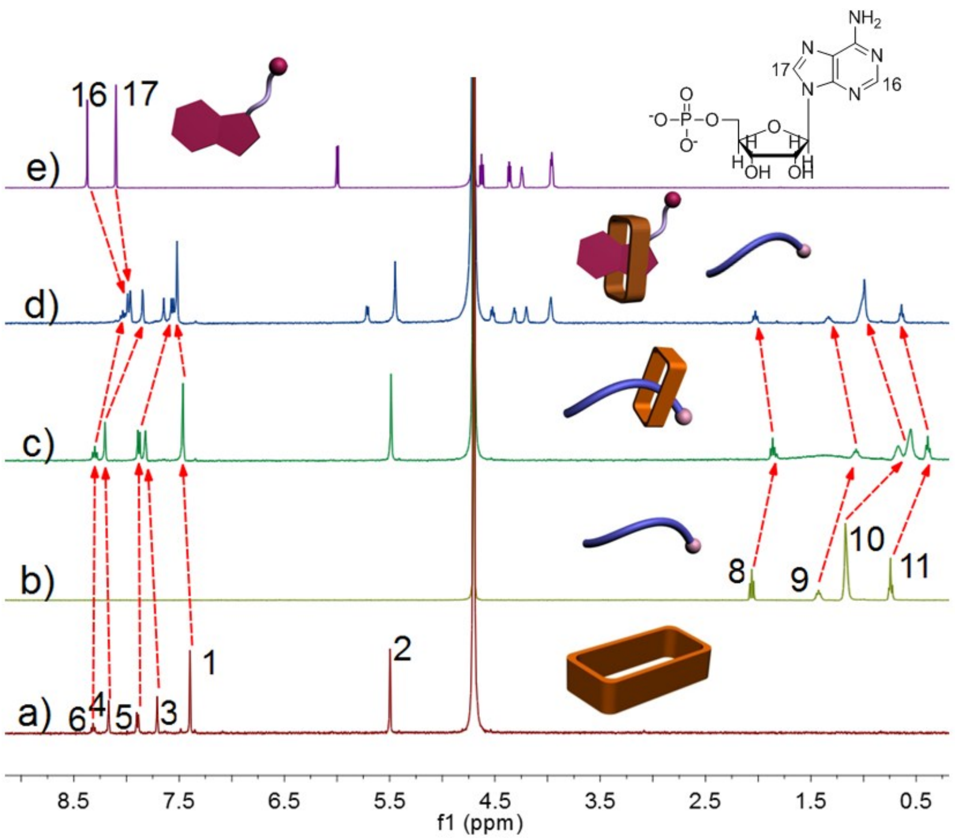

Fig. S21. ^{1}H NMR spectra (500 MHz, D_2O, 298 K): a) 3.00 mM **1**; b) 3.00 mM **2**; c) **1** and **2** (3.00 mM for each); c) **1**, **2**, and AMP (3.00 mM for each); e) 3.00 mM AMP.

8. Nucleotide-induced release experiments

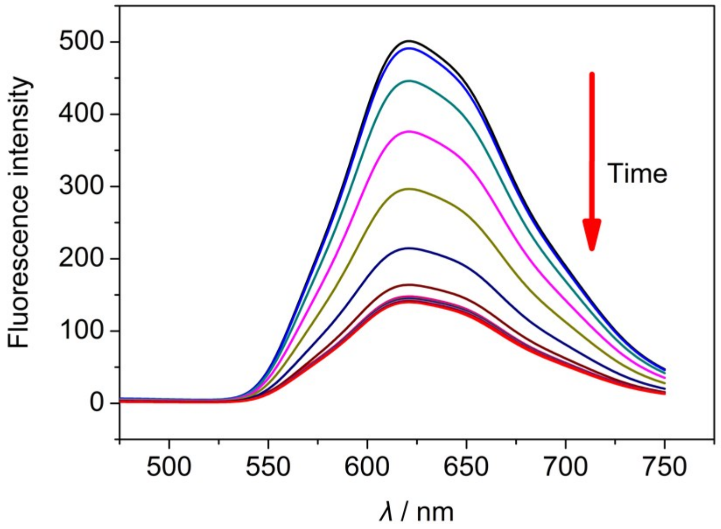

Fig. S22. Changes in the fluorescence spectrum of a solution of micelle (6.50×10^{-7} M **3** and 1.52×10^{-5} M **1**)/DOX (2.24×10^{-7} M) as a function of time (0→28 h) as seen upon exposure to ATP (1.52×10^{-5} M, 1.0 equiv. of **1**) (pH = 7.0, λ_{ex} = 460).

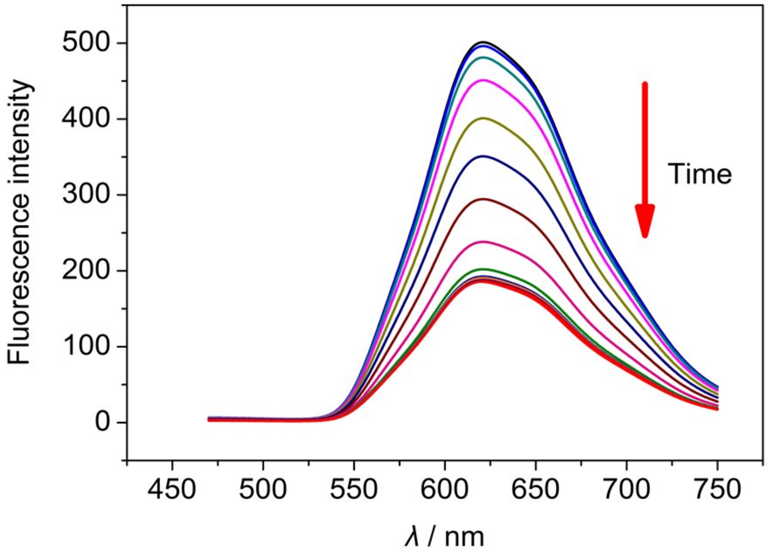

Fig. S23. Changes in the fluorescence spectrum of a solution of micelle (6.50×10^{-7} M **3** and 1.52×10^{-5} M **1**)/DOX (2.24×10^{-7} M) as a function of time (0→28 h) as seen upon exposure to ADP (1.52×10^{-5} M, 1.0 equiv. of **1**) (pH = 7.0, λ_{ex} = 460).

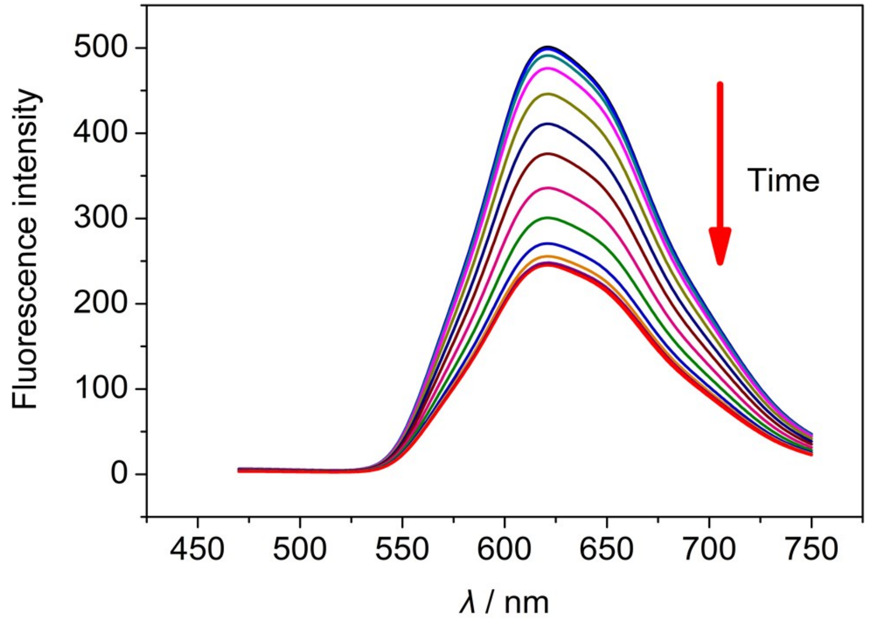

Fig. S24. Changes in the fluorescence spectrum of a solution of micelle (6.50×10^{-7} M **3** and 1.52×10^{-5} M **1**)/DOX (2.24×10^{-7} M) as a function of time (0→28 h) as seen upon exposure to AMP (1.52×10^{-5} M, 1.0 equiv. of **1**) (pH = 7.0, $\lambda_{ex} = 460$).

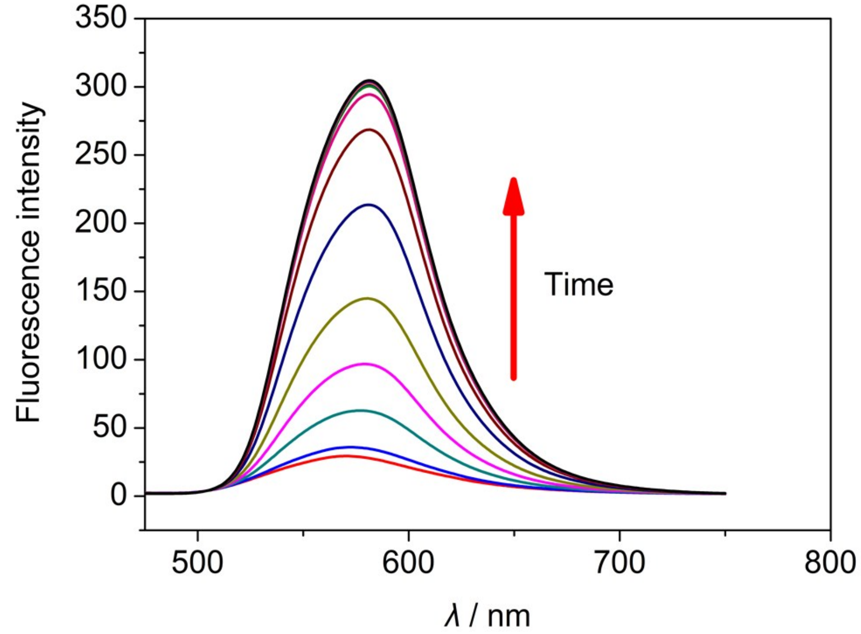

Fig. S25. Changes in the fluorescence spectrum of a solution of vesicle (8.50×10^{-7} M **3** and 4.40×10^{-6} M **1**)/FITC (5.46×10^{-7} M) as a function of time (0→26 h) as seen upon exposure to ATP (4.40×10^{-6} M, 1.0 equiv. of **1**) (pH = 7.0, $\lambda_{ex} = 480$).

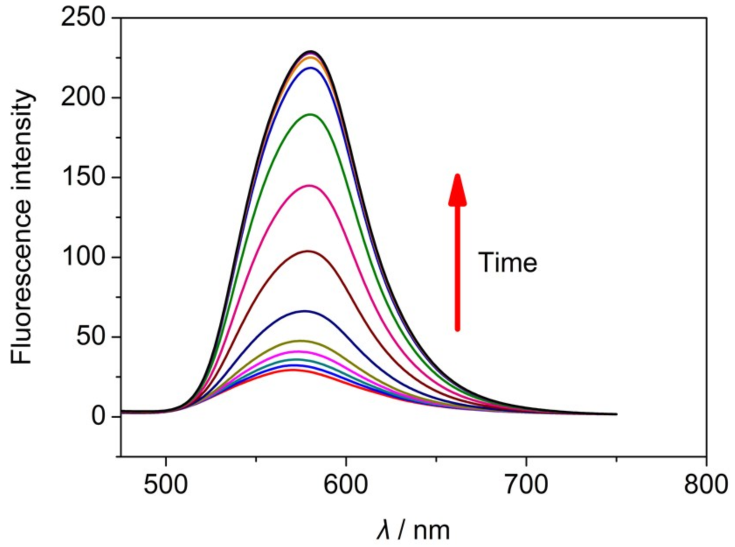

Fig. S26. Changes in the fluorescence spectrum of a solution of vesicle (8.50×10^{-7} M **3** and 4.40×10^{-6} M **1**)/FITC (5.46×10^{-7} M) as a function of time (0→26 h) as seen upon exposure to ADP (4.40×10^{-6} M, 1.0 equiv. of **1**) (pH = 7.0, λ_{ex} = 480).

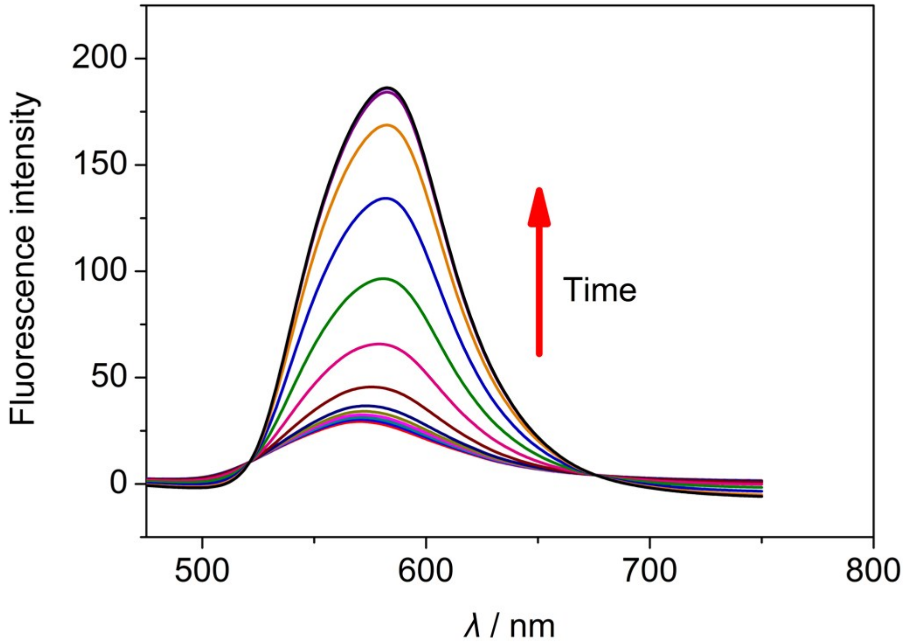

Fig. S27. Changes in the fluorescence spectrum of a solution of vesicle (8.50×10^{-7} M **3** and 4.40×10^{-6} M **1**)/FITC (5.46×10^{-7} M) as a function of time (0→26 h) as seen upon exposure to AMP (4.40×10^{-6} M, 1.0 equiv. of **1**) (pH = 7.0, λ_{ex} = 480).

9. The stability of different aggregates

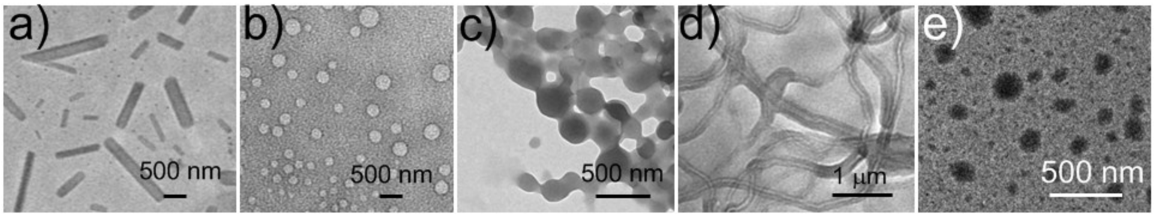

Fig. S28. TEM images of the aggregates after one week, which formed from copolymer **3** (0.100 mM) upon the addition of different amounts of **1** (mM): a) 0; b) 0.500; c) 1.00; d) 1.50; e) 2.30.

10. A TEM image with two types of aggregates

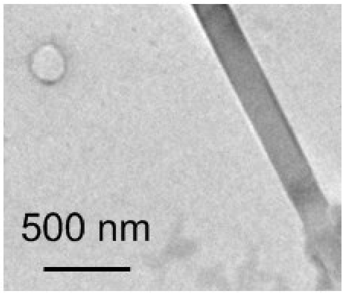

Fig. S29. TEM image of the aggregates formed from copolymer **3** (0.100 mM) upon the addition of **1** (0.300 mM).

11. TEM images after loading DOX or FITC

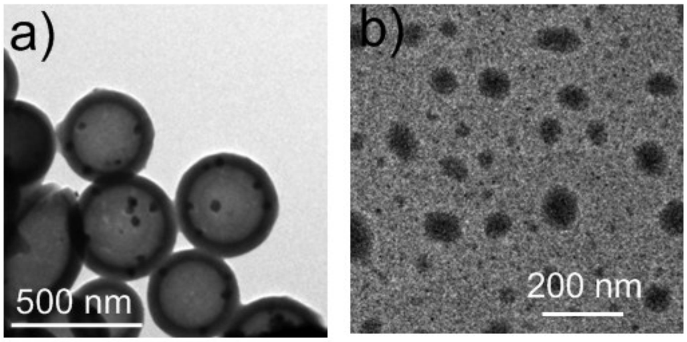

Fig. S30. TEM images: a) FITC loaded vesicles and b) DOX loaded micelles.

12. DLS after loading DOX or FITC

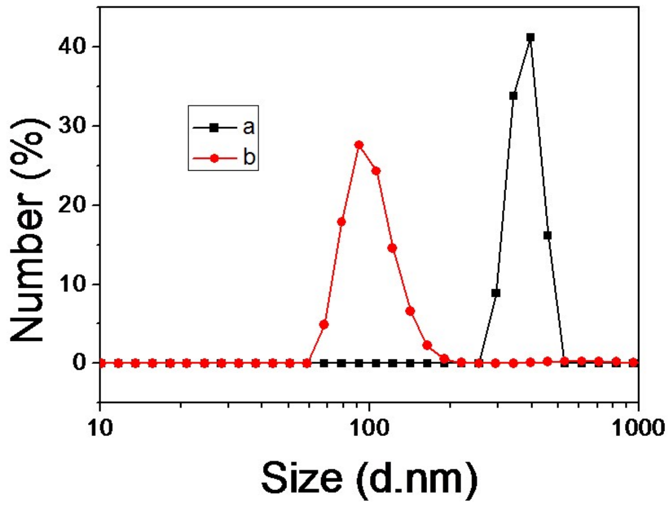

Fig. S31. DLS data: a) FITC loaded vesicles and b) DOX loaded micelles.

13. The drug loading percentages

After dialyzing the mixture of DOX (2.00 mg) and micelle solution (10 mL, 42.00 mg of **3** and 17.70 mg of **1**), 59.90 mg of a red powder was obtained, thus 0.20 mg DOX was loaded in the micelle solution, and the DOX load percentage is 10 wt%. Similarly, the FITC load percentage is 12 wt%.

14. References:

S1. H.-Y. Gong, B. M. Rambo, E. Karnas, V. M. Lynch and J. L. Sessler, *Nat. Chem.*, 2010, **2**, 406–409.

S2. T. Park and S. C. Zimmerman, *J. Am. Chem. Soc.*, 2006, **128**, 11582−11590.

S3. K. A. Connors, Binding Constants; Wiley: New York, 1987. P. S. Corbin, Ph.D. Dissertation, University of Illinois at Urbana-Champaign, Urbana, IL, 1999. P. R. Ashton, R. Ballardini, V. Balzani, M. Belohradsky, M. T. Gandolfi, D. Philp, L. Prodi, F. M. Raymo, M. V. Reddington, N. Spencer, J. F. Stoddart, M. Venturi, D. J. Williams, *J. Am. Chem. Soc.*, 1996, **118**, 4931–4951.
